# Cellular mechanotransduction in health and diseases: from molecular mechanism to therapeutic targets

**DOI:** 10.1038/s41392-023-01501-9

**Published:** 2023-07-31

**Authors:** Xingpeng Di, Xiaoshuai Gao, Liao Peng, Jianzhong Ai, Xi Jin, Shiqian Qi, Hong Li, Kunjie Wang, Deyi Luo

**Affiliations:** 1 Department of Urology and Institute of Urology, National Clinical Research Center for Geriatrics, West China Hospital, Sichuan University, Chengdu, P.R. China; 2 State Key Laboratory of Biotherapy and Cancer Center, West China Hospital, Sichuan University, Chengdu, P.R. China

**Keywords:** Cancer microenvironment, Molecular biology, Cell biology, Respiratory tract diseases, Cancer

## Abstract

Cellular mechanotransduction, a critical regulator of numerous biological processes, is the conversion from mechanical signals to biochemical signals regarding cell activities and metabolism. Typical mechanical cues in organisms include hydrostatic pressure, fluid shear stress, tensile force, extracellular matrix stiffness or tissue elasticity, and extracellular fluid viscosity. Mechanotransduction has been expected to trigger multiple biological processes, such as embryonic development, tissue repair and regeneration. However, prolonged excessive mechanical stimulation can result in pathological processes, such as multi-organ fibrosis, tumorigenesis, and cancer immunotherapy resistance. Although the associations between mechanical cues and normal tissue homeostasis or diseases have been identified, the regulatory mechanisms among different mechanical cues are not yet comprehensively illustrated, and no effective therapies are currently available targeting mechanical cue-related signaling. This review systematically summarizes the characteristics and regulatory mechanisms of typical mechanical cues in normal conditions and diseases with the updated evidence. The key effectors responding to mechanical stimulations are listed, such as Piezo channels, integrins, Yes-associated protein (YAP) /transcriptional coactivator with PDZ-binding motif (TAZ), and transient receptor potential vanilloid 4 (TRPV4). We also reviewed the key signaling pathways, therapeutic targets and cutting-edge clinical applications of diseases related to mechanical cues.

## Introduction

**Fig. 1:**
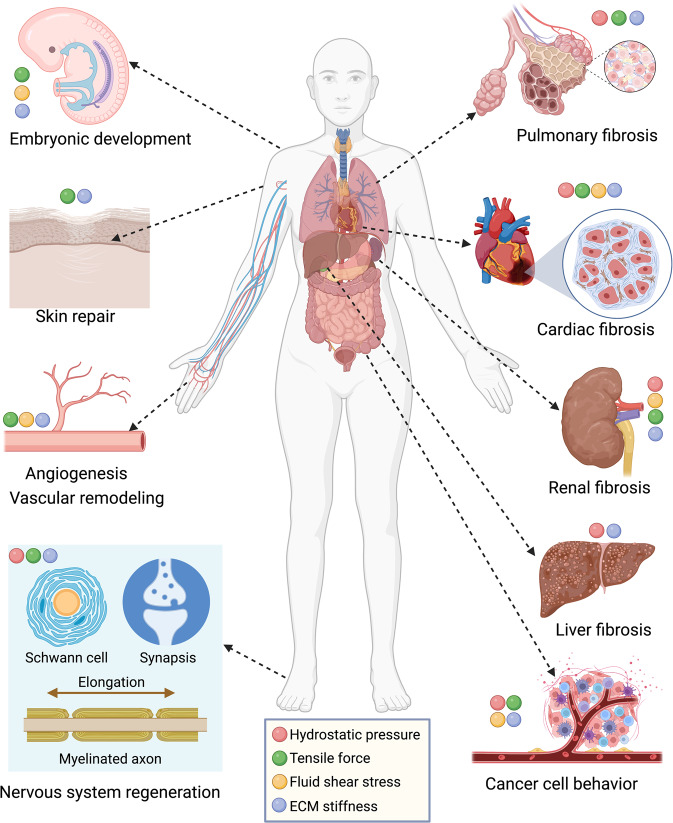
Global overview of the mechanical cue-associated pathophysiological processes. The main components of biomechanical cues engage in several biological processes and diseases, such as pulmonary fibrosis, cardiac fibrosis, renal fibrosis, liver fibrosis, cancer cell behaviors, embryonic development, skin and wound repair, angiogenesis and vascular remodeling, and nervous system regeneration. ECM extracellular matrix. This figure was created using Biorender.com

Cellular mechanotransduction is an important biological process in living organisms. It was first studied based on the Wolff’s Law on mechanical load in tissue homeostasis and has extended to the growth and development of tissues and organisms.^1^ To date, the study of cellular mechanotransduction is being investigated for its impact of multiple mechanical cues on multiple pathophysiological processes, including embryonic development,^2^ tissue repair and wound healing,^3^ neural regeneration,^4^ fibrosis,^5^ tumorigenesis,^6^ and cancer immunotherapy resistance^7^ (Fig. 1). Many ongoing studies are focusing on the mechanism and therapeutic targets for mechanical cue-induced tissue homeostasis, and corresponding diseases.

Biological mechanical cues mainly include hydrostatic pressure (HP), tensile force (TF) or stretching force, fluid shear stress (FSS), extracellular matrix (ECM) stiffness or tissue elasticity, and extracellular fluid (ECF) viscosity. These mechanical cues are involved in the communications in cell–ECM, cell hemodynamics, and cell–ECM–cell crosstalk.^5^ However, the crosstalk between the normal tissue homeostasis and lesions is also closely associated with mechanical stimulation, which restricts the investigation of therapeutic targets. Hence, finding strategies for balancing the mechanical cues in a normal range is of great importance.

Cells can sense the mechanical cues and respond to the changes in the biomechanical environment. The functional ion channels and receptors on the cell membrane can sense the biomechanical signals, and trigger changes in cytoskeleton structure and downstream biochemical signaling cascades.^8^ The nuclear membrane can also sense the alteration of cytoskeleton, thereby influencing downstream gene transcription. An intricate regulatory network of the cellular mechanotransduction process has already been established. These signaling pathways extensively intervene in the pathophysiological process of the human body.

Recent studies on the biological function of cellular mechanotransduction have provided a broader view of the interaction between mechanical cues and biological processes. However, the regulatory mechanisms of mechanical cues are complex and how these mechanical cues interact with each other is still poorly studied. Meanwhile, many signaling pathways have been identified without fully illustrating the cell function alteration, including cytoskeleton swelling and contraction, cell polarization, and cell adhesion. The understanding of cellular response to mechanical cues can assist in determining the precise approach targeting cellular mechanotransduction.

There have been several reviews investigating the communication between mechanotransduction and diseases. Nevertheless, these reviews almost focus on specific mechanical situations, technology tools or diseases, lacking discussion of therapeutic intervention and clinical application. In this review, we intend to summarize the characteristics and molecular mechanisms of cellular mechanotransduction in normal conditions and diseases with an updated and comprehensive interpretation. In addition, the mechanosensitive effectors, signaling pathways and clinical applications targeting mechanical cues-induced diseases are presented in light of the most recent advances.

## Characteristics and mechanisms of cellular mechanotransduction

**Fig. 2:**
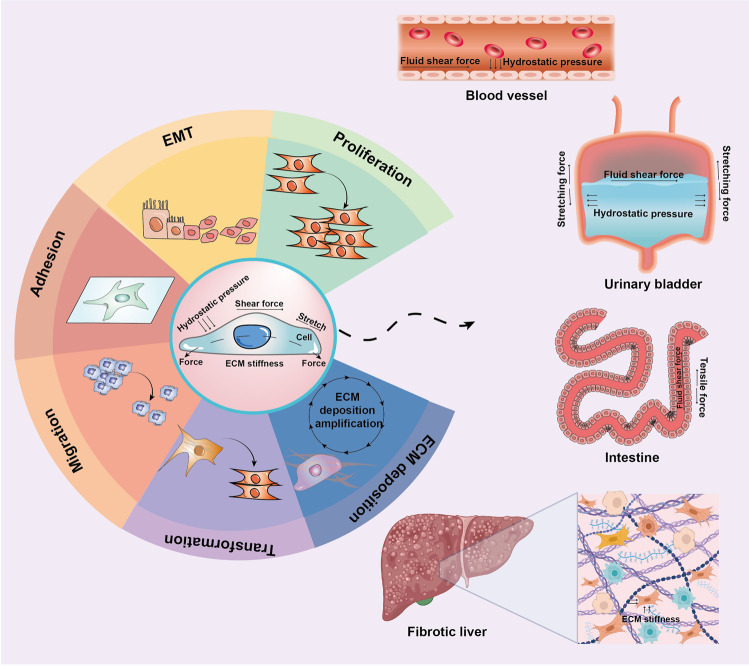
Cellular mechanotransduction in tissues and organs. Typically, fluid shear force and hydrostatic pressure exist in blood vessels. Tensile force, hydrostatic pressure, and fluid shear force function in the urinary bladder. The fluid shear force and tensile force function in the intestine. The ECM stiffness functions in fibrotic liver. ECM extracellular matrix, EMT epithelial–to-mesenchymal transition. This figure was partly created using Biorender.com

HP, FSS, TF, and ECM stiffness are the main biological mechanical cues affecting cell-matrix communications and signal transduction. These mechanical cues regulate multiple biological processes, including cell development, transformation, differentiation, adhesion, migration, proliferation, and ECM generation (Fig. 2). Mechanical cues can also trigger sophisticated biological modulated network-dependent tissue and organ development, regeneration, repair, tumorigenesis, tumor invasion, and metastasis. Herein, we focus on the molecular biology of these mechanical cues.

### Hydrostatic pressure

HP generally exists in tissues and organs with fluids, such as blood vessels, heart, eye, joint cavity, and urinary bladder. The mechanical cues in hollow organs include HP, sinusoidal stress, and interstitial fluid pressure.^9^ HP in the interstitial cavity is approximately −4 cmH_2_O^10^; in solid tumors and edematous tissues, the pressure is 25–40 cmH_2_O^11^; in the liver, an increase of intrahepatic resistance induces augments in the pressure gradient between the inferior vena cava and the portal vein (>5 mmHg).^12^

HP in the normal range, also being classified as physiological HP, can promote the development and repair of tissues. Studies have revealed that periodic HP promotes bone growth and organization in developmental models.^13,14^ However, HP beyond the normal range, also being classified as pathological HP, can lead to decompensated lesions in tissues and organs, such as bladder fibrosis and decreased sperm quality.^15^ Osteogenesis and bone density can also be enhanced by HP.^13^ Additionally, HP can change the conformation of ion channels and regulate ion transmembrane transportation,^16^ thereby affecting pathophysiological processes. For example, HP induces the opening of transmembranal channels in Hela cells, resulting in an influx of calcium ions (Ca^2+^).^17^ As a mechanosensitive ion channel protein, Piezo1 can respond to HP, activate mitogen-activated protein kinases (MAPK) and p38 signaling pathways, and facilitate the expression of bone morphogenetic protein 2 (BMP2) to affect the phenotype of mesenchymal stem cells.^18^ Furthermore, high HP (40 mmHg) promotes atrial electrophysiological remodeling and inflammatory response by regulating the ion flow, reducing atrial fibrillation.^19^

**Fig. 3:**
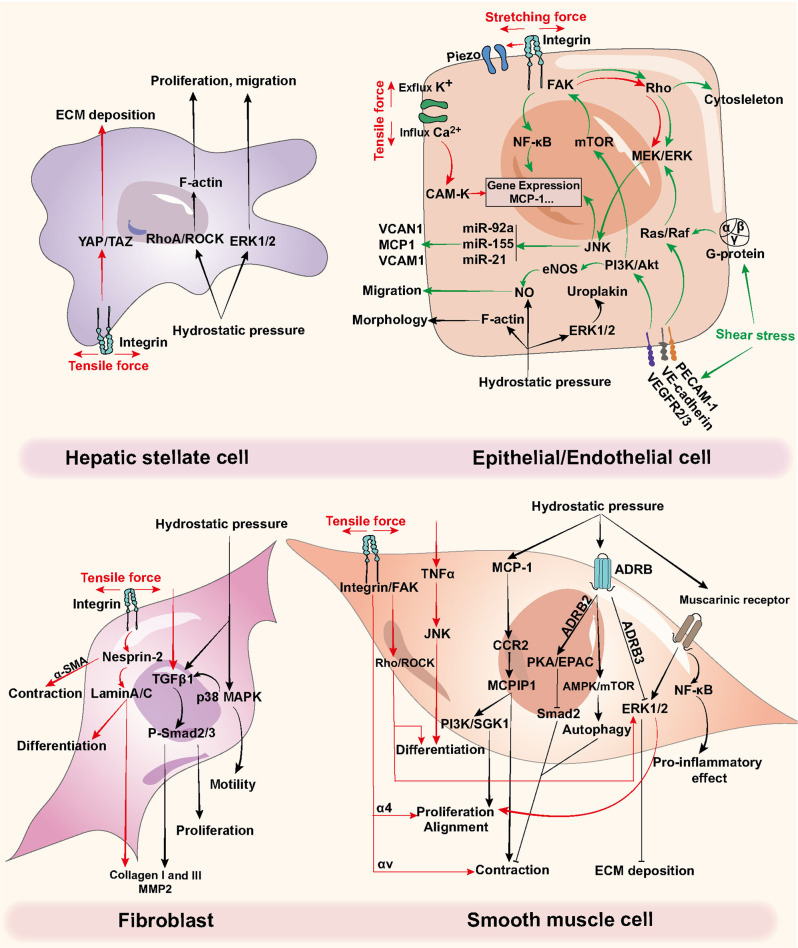
Regulatory mechanisms of tensile force, hydrostatic pressure, and shear stress on different cell types. Red arrows refer to stretching force, green arrows refer to shear stress, and black arrows refer to hydrostatic pressure. ADRB β adrenoceptors, ECM extracellular matrix, ERK extracellular signal-related kinase, MCP1 monocyte chemotactic protein 1, MCPIP1 MCP1-induced protein, MMP matrix metalloprotease, mTOR mammalian target of rapamycin, NF-κB nuclear factor-κB, TGFβ transforming growth factor β, TNFα tumor necrosis factor α, VCAM-1 vascular cell adhesion molecule 1

HP signals transduce through various functional proteins and signaling pathways (Fig. 3). For parenchymal cells such as hepatocytes and hepatic stellate cells (HSCs), the subcapsular HP affects the biochemical processes.^20^ The cytoskeleton-related signals (i.e., RhoA, ROCK, α-SMA) are enhanced by 50 mmHg of HP on HSCs. The increase of interstitial fluid pressure activates the HSCs to facilitate fibrosis progression.^21^ Moreover, in hollow organs such as the urinary bladder, the uroplakins (Ia/Ib/II/III) on the epithelial cell membrane, which play a pivotal role in cell differentiation,^22,23^ are the main components of the high-resistance barrier of urinary bladder urothelium.^24^ Intravesical sustained HP above 40 cmH_2_O is generally known as pathological pressure and is a potential cause of fibrosis.^25^ It has been reported that 200 cmH_2_O HP can facilitate the expression of uroplakin Ia and uroplakin II protein in urothelial cells, which are the key factors for extracellular signal-related kinase (ERK) 1/2 pathway activation.^26^

All the mechanosensitive parenchymal cells and nonparenchymal cells (i.e., fibroblasts and immune cells) serve as effectors responding to HP. For example, endothelin-1 (ET-1) and nitric oxide are increased under HP and identified as novel insights for vascular remodeling.^27^ The M3 muscarinic receptor subtypes are mainly distributed in the neck and dome region of the urinary bladder. M2 and M3 muscarinic receptors are involved in the cholinergic transmission process in the human detrusor muscle or dome.^28,29^ Pathological HP also induces atrial fibroblast proliferation and collagen deposition through the transforming growth factor β1 (TGFβ1)/Smad3 pathway.^30^ Similarly, the motility and proliferation of fibroblast are promoted by increased HP-induced p38 MAPK cascades.^31,32^

### Fluid shear stress

The intra-organ or intra-capsule fluid can both generate FSS by the fluid flow. Typical FSS exists in human vasculature (i.e., vessel bifurcations, stenosis, aortic aneurysms, heart valves, and capillary networks), including shear and extensional flow.^33^ The shear flow is classified as laminar flow and turbulent flow influenced by the structure of the lumen. Uniaxial extensional (elongational) flow is the flow acceleration parallel to the vascular wall. Extensional stress is common in regions with sudden contractions or expansions of fluid flow. Stable flow or laminar flow functions in anti-inflammation, anti-adhesion, and anti-thrombosis in the vascular wall.^34^ However, persistent turbulent flow in the vascular wall can increase the endothelial permeability (i.e., junctional proteins alteration) and proinflammatory signaling (i.e., nuclear factor κB [NF-κB] signaling, adhesion molecules activation) to promote the formation of lesions.^35,36^

FSS determines the tissue homeostasis such as blood vessels, the heart, the airway, and the urinary bladder^37–39^ (Fig. 3). The frictional forces generated by the bloodstream, also known as the wall FSS, can lead to the luminal stress of the vascular wall.^40^ High FSS can induce anti-inflammatory effects, such as Klf2/4 or endothelial nitric oxide synthase (eNOS). The turbulent flow, oscillatory, and low FSS trigger pro-inflammatory responses. In the circulatory system, the bloodstream is generated by the heart contract, which produces FSS. In this situation, FSS is determined by wall shear rate and blood viscosity.^41^ FSS in large blood vessels and arterioles are approximately 10 dyn/cm^2^ and 50 dyn/cm^2^, respectively.^42^ Many studies have shown that FSS facilitates epithelial cells and triggers atherosclerosis, lipid deposition, and vessel wall thickening,^43^ while the endothelial cells remain polygonal in shape and unorganized in turbulent flow or low FSS (≤5 dyn/cm^2^).^44^

The blood flow-induced hemodynamic changes regulate multiple signaling pathways in various vascular wall cells.^45–47^ The recently reported signaling pathways related to the FSS mainly include vascular endothelial growth factor (VEGF), Notch, PDGF, Klf2, eNOS, endothelin, Rho family signaling molecules of TGFβ/BMP/Smad pathway, MAPK signaling pathway, NF-κB signaling pathway, and GTPase signaling pathway.^48,49^ When the cells receive the FSS mechanical signals, several mechanosensors will be triggered, including integrins, the glycocalyx, primary cilia, G-protein-coupled receptors, and ion channels (K^+^, Ca^2+^).^50–57^ Piezo channels are important sensors for mechanical stimulation. Piezo1 initially senses SS and transmits biomechanical signals to the nucleus to promote nuclear contraction.^58^ In response to FSS, several mechanosensory complexes are also activated, including vascular endothelial cell cadherin (VE-cadherin), VEGF receptor 2 (VEGFR2), and platelet endothelial cell adhesion molecule (PECAM-1, or CD31). As a result, the mechanosensory complexes transmit the biomechanical signal into the endothelial cell.^55^ PECAM-1 activates Src, and binds to type III intermediate filament, in which process VE-cadherin serves as an adapter.^59^ In addition, G-protein-coupled receptor is also triggered to enhance Ras and Rho GTPase signaling cascades.^60^ The enhanced Rho activity increased endothelial cell migration through FSS and modulated the traction force.^61^ In addition, PI3K/AKT/mammalian target of rapamycin (mTOR) can also be activated by VEGFR2, which ultimately induces integrin activation.^62^ A recent study concluded that β integrin, a specific sensor of unidirectional FSS but not bidirectional, drives the endothelial cell alignment and downstream cascade.^63^ The integrin/NF-κB-associated cascades and adhesion of endothelial cells are initiated by FSS.^64^ Furthermore, the long-term presence of NF-κB and other inflammatory molecules increases the expression of intercellular adhesion molecule (ICAM-1) and vascular adhesion molecule (VCAM-1), further recruiting monocytes to aggravating epithelial inflammation and inducing atherosclerosis under FSS.^65^ SH2-containing protein tyrosine phosphatase-2 (SHP-2) has been reported to bind to PECAM-1 to activate the ERK1/2 cascades.^66^ In the hydrodynamic model, the carbon monoxide synthesis increases, and endothelial cell structure is remodeled by FSS.^27^ It has also been reported to promote endothelial cell migration through Tie2/PI3K/AKT/eNOS pathway.^67^

Apart from the functional proteins and signaling pathways, noncoding RNAs (ncRNAs) also serve as sensors of FSS. ncRNA is essential in the gene-regulatory process by pairing to the mRNAs and is composed of small and long noncoding RNAs.^68^ In addition, the response of ncRNAs to biomechanical signal is involved in physiological changes in atherosclerosis, atherogenesis,^69^ various cardiovascular diseases,^70^ and coronary syndromes.^71–73^ Similarly, some small RNAs also engage in mechanotransduction to promote diseases progression. Small RNAs include microRNAs (miRNAs), PIWI-interacting RNAs (piRNAs), and short-interfering RNAs (siRNAs).^74^ Some miRNAs have been identified as mechanical cues-induced reactions, including miR-10a, miR-19a, miR-23b, miR-101, and miR-143/145.^75,76^ For example, atherogenesis is promoted by mechanical stimuli-induced miRNA-associated signaling pathways in endothelial cells. Several miRNAs have been illustrated in atherosclerosis, such as miR-17-3p, miR-92, miR-126, miR-712, miR-205, miR-143, miR-145, miR-31, and others.^77^ Novel therapeutic approaches targeting ncRNAs and miRNAs are necessary for disease treatment.

### Tensile force

TF, also known as stretching force, is important in muscle and joint movement, atherogenesis, cardiovascular remodeling,^78,79^ and cell behaviors (i.e., proliferation, transformation, and development).^80^ For instance, the dynamic tension of the joint can affect the final behaviors of muscles.^81^ In the cardiovascular system, endothelial cells, smooth muscle cells, and cardiac myocytes are the principal effector cells responding to TF generated by blood flow. TF can induce cardiomyocyte hypertrophy through nuclear factor-like 2 (Nrf2) and interferon-regulated transcription factors in myocardial tissue.^82^ In the vascular wall, TF promotes vascular remodeling and contraction.^83^ Besides, TF can induce urothelium proliferation through α6-focal adhesion kinase (FAK) signaling.^84^ It is also a factor leading to the development of animal neurons by regulating gene transcription.^85,86^ Hence, a comprehensive interpretation of the TF in the human body is of great significance.

The magnitude, frequency, and duration of the TF can affect the alignment, differentiation, migration, proliferation, survival, apoptosis, and autocrine and paracrine functions of cells (Fig. 3). Once the integrin receives the biomechanical signals, it will transduce the signals into cells. Then the p38MAPK signaling, NO, and reactive oxygen species (ROS) are activated to trigger downstream cascades.^87,88^ Meanwhile, the α-smooth muscle actin (*α-SMA*) expression and promoter activities are enhanced through JNK and p38MAPK signaling pathways.^89^ Rho also responds to integrins to activate ERK signaling.^90^ Ultimately the phenotype and alignment of cells are regulated.

The nuclear envelope is also an important effector of cellular mechanotransduction. The nucleus membrane senses TF and reacts more rapidly than common biochemical transduction. The interactions between the nuclear envelope and actin have been reported in nuclear migration,^91^ cell polarization,^92^ nuclear deformation,^93^ Piezo1/endoplasmic reticulum (ER) response,^94^ chromosomes gathering,^95^ and chromatin organization.^96^ Significantly, lamins located in the nuclear envelope can form interacting meshwork with a highly branched structure^97,98^ and modulate gene transcription, DNA replication, and chromatin organization.^99^ Lamins also regulate the nucleus assembly, nuclear shape, stiffness, and structure in cytoskeleton arrangement.^100,101^ The nesprins interact with laminA/C to transmit biomechanical signals into the nucleus. When Nesprin2 is inhibited, the cell transformation process and collagen synthesis are blocked.^102^ During this process, the mechanosensitive pathways in mature tissues do not respond to intercellular tension. Thus, these signaling pathways do not influence fibroblast differentiation in subsequent fibrosis.^103^ It is worth noting that different TF intensities induce various effects on the chondrocyte mechanotransduction process. Mechanically activated ion channel transient receptor potential vanilloid 4 (TRPV4)-mediated Ca^2+^ signaling is significant in response to the physiological strain levels (3% and 8%). However, Piezo2-mediated Ca^2+^ signaling is substantial in response to pathological high strain levels (18%).^104^

Like FSS, miRNAs are involved as post-transcriptional regulators in the bone remodeling process and function in cell differentiation in the orthodontic tooth movement process. For instance, it has been confirmed that the miRNA-21 deficiency inhibits orthodontic tooth movement and promotes the remodeling of bone in mice.^105^ Cyclic stretch activates alkaline phosphatase (ALP) and osteogenesis biomarkers such as osteopontin (OPN), runt-related transcription factor 2 (RUNX2), type I collagen, ALP, osteocalcin, and osterix.^106^ The ALP activity is inhibited by miR-146a and miR-34a targeted CUGBP Elav-like family member 3 (*Celf3*), which can regulate the life cycle of mRNAs from transcription to translation.^106–108^

### ECM stiffness

ECM, a non-cellular component presented in extracellular capsules, is a complex that mainly consists of collagen, fibronectin, elastin, lamin, proteoglycan, glycoprotein, and glycosaminoglycan.^109^ Fibrillar proteins (i.e., collagens) are featured by high tensile strength but low elasticity. However, elastic fibers (i.e., elastin) are featured by high elasticity and low tensile strength. Therefore, the components of ECM greatly affect the tissue mechanical properties.^110^

ECM components are critical in cell growth, differentiation, and apoptosis. The cells communicate via the intracellular skeleton and cell surface adhesion molecules with the ECM and neighboring cells. These properties of ECM are also engaged in the occurrence and development of diseases. ECM remodeling is associated with complicated physiological conditions. Pathological ECM has a prolonged impact on the morphology and functions of cells and forms an amplification effect to strengthen the ECM deposition process further, resulting in severe fibrosis.^111^ Excessive deposition of ECM can lead to even worse results. For example, myocardial fibrosis is developed from the fibrotic scar due to myocardial infarction and matrix deposition in the interstitial and perivascular areas. It causes heart function impairment and accelerates the progression of heart failure.^112^ The available evidence demonstrated a predominant increase in type I and type III collagen fibers observed in hypertensive heart disease and aortic stenosis-associated heart failure.^113,114^ Moreover, liver fibrosis has been indicated to be triggered by myofibroblasts from chronic hepatotoxic injury (i.e.,hepatitis HBV or hepatitis HCV, alcohol abuse) or cholestatic injury (i.e., bile duct obstruction). The HSCs-secreted ECM promotes transformation from fibroblasts to myofibroblasts.^115,116^ As a result, the normal liver tissues were replaced by the cross-linked type I and type III collagen fibers.

The mechanical properties of ECM are also critical in biological processes. ECM stiffness indicates the resistance to the deformation of tissue and induces an increase in tissue elasticity. Apart from the normal deposition of ECM, post-translational modifications of ECM components also modulate stiffness. For instance, the nonenzymatic glycation and cross-linking of collagen can increase the elasticity of the matrix. The stiffness of tissues varies in different organs. For example, normal lung tissues have Young’s Modulus of approximately 1 kPa required for respiration.^117^ However, lung elasticity increases to 30–50 kPa in idiopathic pulmonary fibrosis (IPF) patients due to excessive ECM deposition and contractile myofibroblasts transformation.^118^ Besides, the normal liver tissue represents a stiffness of 1.5–4.5 kPa, and that in the early and late stages of liver fibrosis are 4.1–12.9 and 16.3–48 kPa, respectively.^119–121^

ECM stiffness is involved in multiple pathophysiological processes. In the central nervous system, neurons tend to live in an environment with soft stiffness while stiff substrates are required by glial cells.^122,123^ In addition, pathological ECM stiffness triggers multiple organ fibrosis,^64,124,125^ vascular smooth muscle disorders,^126^ benign pancreatic diseases,^127^ osteoblast differentiation,^128^ tumor development, invasion and metastasis.^129–134^ Studies have shown that cancer-associated fibroblasts (CAFs) can change the composition and physicochemical properties of ECM, thereby regulating tumor metastasis and adjusting the metastasis path.^135,136^

**Fig. 4:**
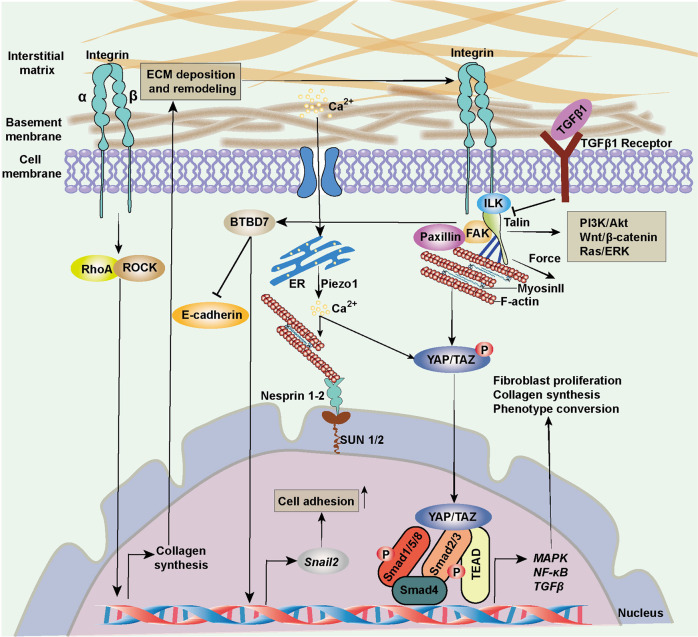
Cellular mechanotransduction of ECM stiffness. The integrins convey mechanical and biochemical signals from ECM into cells and facilitate cell proliferation, differentiation, migration, and invasion. The RhoA/ROCK pathway is activated, enhancing collagen and fibronectin accumulation. Talin/FAK facilitates the assembly of F-actin to promote signal transduction. The actin connects with myosin II and conveys the mechanical cues to the nucleus. YAP/TAZ is translocated into the nucleus to promote the transcription of downstream genes, collagen synthesis, and cell differentiation. ECM extracellular matrix, ER endoplasmic reticulum, ERK extracellular signal-related kinase, FAK focal adhesion kinase, ILK integrin-linked kinase, P phosphate, TGFβ transforming growth factor β

Mechanistically, increasing tissue elasticity activates the integrins, which belong to the cell surface receptors family, to enhance cell-cell communication (Fig. 4). The integrins are abundant in fibroblasts and can receive the signals from ECM components, convey mechanical and biochemical signals into cells, and facilitate cell proliferation, differentiation, migration, and invasion.^137–140^ Once the integrins are activated, the RhoA/ROCK pathway is initiated, enhancing collagen and fibronectin accumulation.^141,142^ The transcriptional regulator BTBD7 is activated to trigger epithelial-to-mesenchymal transition (EMT) through Snail2 while E-cadherin is inhibited, and hence the cell adhesion is weakened.^143^ Meanwhile, talin binds to the cytoplasmic tail of the β integrin and facilitates the assembly of F-actin to promote signal transduction.^138,144,145^ The FAK also interacts with integrins and triggers downstream cascades in fibroblast phenotype conversion.^146,147^ The integrin-linked kinase (ILK) and paxillin have also been reported to be essential linkages of integrin-mediated fibrosis.^148^ The actin connects with myosin II and conveys the biomechanical signals to the nucleus. In this process, Yes-associated protein (YAP) and transcriptional coactivator with PDZ-binding motif (TAZ) are translocated into the nucleus to promote transcription of downstream genes in cell proliferation, collagen synthesis, and cell differentiation. This action inversely enhances ECM stiffness. Significantly, YAP/TAZ-mediated ECM stiffness-induced biochemical transduction is independent of Hippo signaling cascades and the large tumor suppressor kinases.^149^ TGFβ, Wnt/β-catenin, MAPK/ERK, and NF-κB signaling pathways also engage in this process.^150–152^ For example, in liver cancer cells, ECM stiffness can activate TGFβ by regulating the cytoskeletal tension induced by the integrin-FAK-Rho GTPase pathway.^133^ In addition, ECM stiffness regulates mesenchymal stem cell differentiation through the Wnt/β-catenin signaling pathway.^153^ Reducing ECM stiffness can promote M2 macrophage activation and enhance peroxisome proliferator-activated receptor gamma expression.^154^ And pathological ECM stiffness can up-regulate Ca^2+^ ion channels, improve intracellular Ca^2+^ concentration, and ultimately activate the ERK signaling pathway to promote cell proliferation and vascular remodeling.^155^ Intriguingly, a recent study reported that the curved nanofibers in the ECM microenvironment facilitated cell proliferation and osteogenic differentiation.^156^ Differing from a straight fiber network in forming continuous adhesion, a curved fiber structure tends to trigger discrete adhesion. Therefore, the cell bridge forming function of curved matrix supplements the current research in ECM stiffness.

The mechanical cues are transduced through the cytoskeleton in a much faster manner than that through biochemical signaling. The nuclear envelope connects with the actin between the cytoskeleton and nucleoskeleton.^157,158^ The transcriptional alteration better responds to nucleoskeleton change with the assistance of lamins, emerin, and transcriptional regulators.^159–161^ The modifications of ECM and cytoskeleton trigger the mechanotransduction in the cytoplasm and nucleus network to proceed regulation of cell function.^162^

### ECF viscosity

A recent study identified a novel mechanical cue, ECF viscosity, which could interact with ECM stiffness to induce cell migration and substrate mechanotransduction. The macromolecules in the crowded ECF environment determined the density, osmotic pressure, and viscosity. Intuitively, high ECF viscosity declines the motility of various cell types. However, Konstantopoulos et al. found that cell migration and cancer dissemination are facilitated by increased ECF viscosity.^163^ The crosstalk between ECF and cells induces the actin-related protein 2/3 (ARP2/3)-complex-dependent actin network, which triggers Na^+^/H^+^ exchanger 1 (NHE1) polarization. NHE1, an isoform of NHE, is a membrane transporter that exchanges intracellular proton for extracellular Na^+^.^164^ The activation of NHE1 promotes cell swelling and increases cell tension, then facilitates TRPV4-mediated Ca^2+^ influx. Subsequently, RhoA-dependent contractility is increased, thereby enhancing the motility of cells. High ECF viscosity increases the motility of cells on two dimensional (2D) surfaces. ECF viscosity has also been confirmed to promote cell spreading dynamics based on integrin/YAP signaling.^165^ High ECF viscosity triggers integrin-based adhesion, enhancing cell migration. ECF can also interact with ECM stiffness to improve cellular mechanotransduction. Despite these, the mechanisms of the impact of ECF viscosity on cells are largely understudied. The recent studies have provided a highlight for future focus on the mechanosensing process.

In the ECM microenvironment, the cells interacting with a reconstituted three-dimensional (3D) environment have a lower level of actin-associated filamin and tension than that with a 2D system, which unfolds the filamin and interacts more with ER.^166^ Moreover, a rigid 2D substrate induces more integrins than a 3D matrix.^167^ Although the 2D system simulates ECM mechanical cues to a large extent and has long been recognized as a classical foundation,^168^ the 3D system with more compliable components is still preferred for research in the future. The mechanical cues function synergistically or organ-specifically in various all stages of diseases. Therefore, investigating mechanical cues in normal conditions and diseases is essential and provides insights for therapies targeting multiple diseases.

## Critical effectors in mechanical signaling

Mechanical effectors have been widely investigated in recent years, mainly including ion channels,^169–178^ receptors,^179–181^ integrins,^182,183^ and transcriptional factors.^184–187^ These sensors behave differently in the mechanotransduction process and some mechanisms are still poorly elaborated. They function dependently or cooperatively and the interactive networks are sophisticated in regulating cell function. Hence, several typical and highlighted sensors are depicted in the current review.

### Piezo channels in mechanotransduction

Piezo proteins (Piezo1 and Piezo2) are mechanosensitive ion channels encoded by two genes at chromosomes 16 and 18 in humans. Piezo proteins are initially identified as evolutionary conserved mechanically activated cation channels, producing the most stable current under mechanical stimulation.^188,189^ Human transmembrane Piezo1 consists of 2521 amino acids, and Piezo2 consists of 2752 amino acids with a molecular weight of about 300 kDa.^190,191^ The Piezo channels with three kinetic states (open, closed, and inactivated) are responsible for converting the mechanical cues into biochemical signals.^192,193^ It has been proved that Piezo1 senses bilayer tension in bleb membranes that can be modulated by cytoskeletal proteins and ECM stiffness.^194–196^ Piezo1 selectively conducts cations including K^+^, Na^+^, Mg^2+^, and especially Ca^2+^, while Piezo2 indicates non-selective properties in ions conduction.^188,197^

Piezo channels function in the cardiovascular, gastrointestinal, nervous, respiratory, exercise, and urinary systems of the human body^198–225^ (Table 1). Piezo channels have been reported to be related to several pathophysiological processes, including erythrocyte volume regulation,^201^ cell division,^226^ cell migration,^227^ and innate immunity.^228^ Piezo channels are presented in various mechanically sensitive cells coupling Ca^2+^ transmembrane conversion.^229^ In addition, Piezo1 is mainly associated with Ca^2+^ signaling, and Piezo2 functions in touching sensation, tactile pain, balance, respiration, blood pressure, and urinary bladder fullness.^218,230–232^ For instance, the ablation of *Piezo2* results in impaired bladder filling in humans and mice.^221,225^ In the innate immune system, immune cells respond to the local environment and physiological changes, including temperature, pH, oxygen, and nutrition. Mechanical cues are highlighted as contributing factors of immune cell activation.^228^ In the orthodontic tooth movement mice model, Piezo1/AKT/Ccnd1 is essential in bone marrow-derived macrophage proliferation.^233^

**Table 1:** Functions of Piezo channels in cellular mechanotransduction

Type	Target	Mechanical stimulation	Mechanism	Reference
Piezo1	Vascular endothelium development	Shear stress	Ca^2+^influx→MTP-MMP signaling→focal adhesion and endothelial cell sprout formation;	^232^
	Vascular tone	Shear stress	Ca^2+^influx→G-coupled endothelial adrenomedullin receptor→cAMP→ eNOS→NO; Ca^2+^influx→ATP→PI3K/AKT→eNOS→NO	^198–200^
	Vascular remodeling	Stretch	Ca^2+^influx→transglutaminase →ECM remodeling	^222^
	Erythrocytes	Shear stress	Ca^2+^influx→K^+^ efflux→red blood cells dehydration	^201^
	Erythrocytes	Shear stress	Ca^2+^influx→pannexin-1→ATP release	^202^
	Nervous system	Traction force	Ca^2+^influx→neural differentiation →neuron-astrocyte interaction	^203,204^
	Gastric mucosa	Antrum distension	Activated G cells→gastrin secretion	^205^
	Lung endothelium	Shear stress	Ca^2+^influx→calpain→Src cleavage→stabilization of adherens junctions	^206^
	Lung endothelium	Hydrostatic pressure	Ca^2+^influx→calpain→disruption of adherens junctions	^207^
	Aoveoli	Stretch	Ca^2+^influx→Bcl-2 pathway→type II epithelial cells apoptosis	^208^
	Urinary bladder	Stretch	Ca^2+^influx→ATP→attenuate storage disorders	^209^
	Tumor	ECM stiffness	YAP-Piezo1→proliferation; Ca^2+^influx→AKT/mTOR phosphorylation→proliferation; Piezo1–mitochondrial calcium uniporter–HIF-1α–VEGF axis→metastasis	^210,211,213^
Piezo2	Gastrointestinal epithelium	Mucosal force	5-HT pathway→mucosal secretion	^212^
	Airway	Stretch	Ablation of *Piezo2*→Airway-innervating sensory neurons→ respiratory distress and death in newborn mice	^214,215^
	Urinary bladder	Stretch	Sensory neuron→bladder filling sensation	^221^
Piezo1/2	Baroreceptor reflex	Shear stress	Elevated blood pressure→Piezo1/2 →nodose-petrosal-jugular-ganglion complex→ decreased blood pressure and heart rate	^216–218^
	Chondrocyte anabolic and biosynthesis	Mechanical stress	GsMTx4→Piezo1/2 inhibition→ alleviate chondrocyte injury	^219,220^

*ECM* extracellular matrix, *MMP* matrix metalloprotease, *5-HT* 5-hydroxytryptamine

Many mechanical cues behave based on Piezo channels, such as ultrasonic stimulation,^234^ mechanical pulling,^235^ and fluid flow.^222,236^ For instance, the Piezo1 channel expresses in endothelial cells and is involved in vascular development and vascular tone.^232,237^ However, the increase in blood pressure of a *Piezo1* disruption mice model contradicts the results above.^238^ Piezo1 triggers the elevation of blood pressure in the body-activated state instead of the body-inactivated state. In addition, the central nervous system can detect mechanical cues that modulate cell differentiation, cell migration, cell adhesion, gene expression, ion conversion, vesicular transportation, and fluid homeostasis.^239,240^ Mechanical stretch can also induce apoptosis in dense cell regions and cell differentiation in sparse cell regions through Piezo1.^226^ The fundamental senses, such as touch, proprioception, and mechanical pain, are closely associated with Piezo2 channels.^241–245^ In glaucoma patients, ECM deposition has been detected in the trabecular meshwork.^246^ The activation of Piezo1 by HP triggers ECM degradation and suppresses fibronectin synthesis.^247^ However, some contradicting results showed that stretching force induces human trabecular meshwork cells’ ECM production. This is possibly due to the alleviation of the effect of mechanical force by Piezo1 activation. TF and ECM stiffness regulate Piezo1 activation to different degrees. Further investigations are required on how Piezo channels mediate mechanical cues-induced diseases and which pathway plays a predominant role in disease progression. Pharmacological modulators can also activate Piezo channels. For instance, Jedi1/2 and Yoda1 can trigger Piezo1 independent of mechanical cues.^248,249^ In the urinary system, Piezo1 increases in the partial bladder outlet obstruction model, contributing to bladder injury. The inhibition of Piezo1 might be an alternative therapy to ameliorate bladder storage dysfunction.^250^ In addition, Piezo1 and Piezo2 often function synergistically. Piezo2 is reported to engage in the bladder filling process, and lack of Piezo2 in sensory neurons led to bladder dysfunction to some extent. However, the ablation of *Piezo2* in mice does not cause complete urinary tract function loss, and Piezo2-deficient humans are still able to urinate.^221^ The co-function of Piezo1 in mediating stretching responses might be the reason.

### Integrins in mechanotransduction

Integrins are identified as transmembranal receptors on the cell surface that function in cell adhesion and biochemical signal transduction. In mammals, there are a total of 24 different pairs of heterodimeric integrins comprising 18 α and 8 β subunits.^251,252^ In general, the cytoplasmic domain of many β subunits is highly homologous, and the integrins bind cytoskeletal structures through β subunits.^253^ The integrin complexes express cell-specifically depending on the cell type and developmental stage. Different integrin complexes function in various phenotypes. For instance, the integrin αvβ6 has been identified to trigger spatially restricted activation of TGFβ1 to prevent fibrosis progression.^254^ In both humans and animals, the loss of integrin v6 activity can lead to baldness, amelogenesis imperfecta, periodontal disease, and altered immunological responses in the lungs and skin.^255^ In contrast, α1β1, α5β1, and α7β1, which function in collagen, fibronectin, and laminin-binding receptors, are highly expressed in the cardiomyocyte to promote fibrosis.^256–258^ Besides, cardiac fibroblast expresses α1β1, α2β1, α11β1, and β3 integrins, which act as collagen-binding integrins.^259–262^ The integrin αv is recognized as a principal pathway in multiple organ fibrosis. The attenuation of integrin αv can alleviate liver and lung fibrosis.^263^ The integrins α1β1, α2β1, α3β1, α4β1, α5β1, α6β1, αvβ1, αvβ3, and αvβ5 have all been identified in cardofibroblast that play important role in cardiac remodeling. Apart from the above statement, the integrins α8β1 and α11β1 are selectively expressed HSCs that induce liver fibrosis.^264,265^

**Fig. 5:**
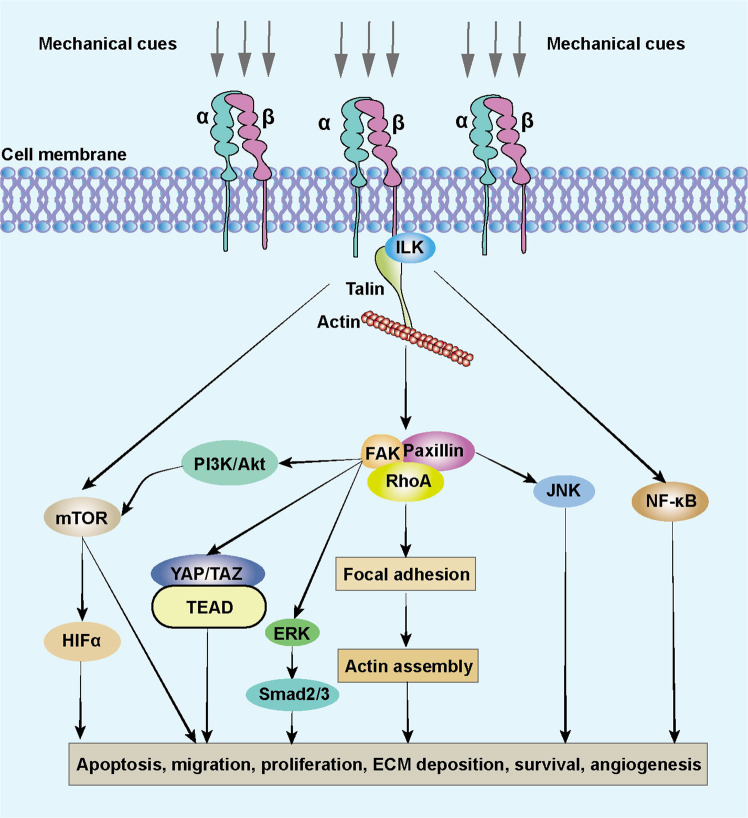
Mechanisms of integrins responding to mechanical stimulation. The β integrin interacts with ILK and talin to trigger downstream cascades. ECM extracellular matrix, ILK integrin-linked kinase

Integrins are also recognized as mechanotransducers in cell-ECM and cell-cell communication processes.^258,266^ Integrins can respond to various mechanical cues, especially the ECM stiffness (Fig. 5). Integrin signaling is assisted by many signaling molecules, including talin, paxillin, vinculin, FAK, ILK, and α-actin, to form links between intracellular and extracellular signals.^267,268^ Integrin-associated signaling pathways are activated by mechanical cues to promote cell proliferation, cell apoptosis, cell migration, cell survival, angiogenesis, and ECM deposition with positive feedback. For example, matrix crosslinking can enhance the integrin signaling cascades to facilitate tumor progression and tissue fibrosis.^269,270^ In renal fibrosis, ECM deposition and cross-linking result in pathological fibrotic change induced by mechanical stress.^271^ The integrin αvβ3 serves as a mechanosensor to promote the development of keloid.^272^ A stiffening keloid matrix activates integrins to induce gene expression through FAK/ERK signaling pathway and interacts with TGFβ/Smad cascades as well.^273^ In cellular mechanotransduction process, integrin-dependent RhoA/ROCK signaling is required in signal transduction and the related gene expression of fibrosis. Besides, RhoA/ROCK also triggers nonmuscle myosin II activity that influences the architecture of cytoskeleton. TF triggers integrins to interact with FAK and Src family kinases and promotes cell stiffening.^274^ The change of cytoskeleton transduces the signal to activate YAP/TAZ dephosphorylation and translocate into the nucleus to promote downstream gene expression through FAK, Src, and JNK signaling pathways.^275–277^

Taken together, integrins are important sensors in mechanotransduction, which can interact with many functional domains and signaling pathways to promote pathophysiological processes. Given that many emerging therapies targeting integrins (i.e., α4β7, αvβ6, and α5β1) in multiple diseases are in process (Table 2), whether the interventions affect normal tissue or organs is uncertain. Therefore, more tissue- or organ-specific therapies targeting integrins are needed. In addition, how these integrins communicate with other receptors, ion channels, or transmembranal proteins still needs further discussion.

**Table 2:** Typical clinical trials targeting integrins

Integrin subtype	Intervention/treatment	Disease type	Phase	Current status	ClinicalTrials.gov identifier
α5β1	Volociximab	Metastatic renal cell carcinoma	2	Terminated	NCT00100685
		Pancreatic cancer	2	Completed	NCT00401570
		Ovarian cancer, primary peritoneal cancer	1/2	Completed	NCT00635193
α4β7	Vedolizumab	Ulcerative colitis	4	Recruiting	NCT05481619
		Crohn’s disease; ulcerative colitis	Not applicable	Completed	NCT02862132
		Inflammatory bowel disease	Not applicable	Completed	NCT02712866
		Type 1 diabetes	1	Recruiting	NCT05281614
αvβ1; αvβ3; αvβ6	IDL-2965 oral capsule	Idiopathic pulmonary fibrosis	1	Terminated	NCT03949530
αvβ6; αvβ1	PLN-74809	Idiopathic pulmonary fibrosis	2	Completed	NCT04072315
αLβ2; α4β1	7HP349	Solid tumor	1	Completed	NCT04508179

### YAP/TAZ in mechanotransduction

YAP was previously isolated from the interacting protein of Yes kinase^278^ and acted as a transcriptional coactivator.^279^ In general, YAP binds to TAZ to interact with the TEAD family of transcriptional enhancers (TEAD1-4) to regulate gene transcription.^280^ YAP functions in tissue and organ development, cell migration, tissue regeneration, and homeostasis.^281^ YAP receives both biochemical and biomechanical signals dependent or independent of the Hippo signaling pathway. Once YAP is activated, it is dephosphorylated and translocates into the nucleus to regulate downstream gene expression. As a transcriptional coactivator, YAP often functions with the assistance of several transcriptional factors, such as Smad, RUNX, signal transducers and activators of transcription (STAT), and others.

As a critical downstream effector of the Hippo signaling pathway, Hippo/YAP signaling pathway regulates the regeneration of organs and induces the occurrence and progression of tumors and fibrotic diseases.^282–285^ For instance, in gastric cancer, Hippo/YAP promotes cancer cell survival and metastasis by activating SIRT/Mfn2/mitochondrial signaling.^286^ Current studies have shown that ECM is one of the crucial factors promoting fibrosis.^287,288^ ECM stiffness can significantly enhance matrix deposition, cell adhesion, and tissue remodeling.^118,289^ YAP/TAZ is highly expressed in fibrotic tissues and upregulates the expression of ECM-related genes through TGFβ/Smad signaling pathway.^151^ High expression of YAP in adherent cells promotes normal cell proliferation in vitro.^290^ However, the excessive dephosphorylation of YAP results in uncontrolled cell proliferation and, ultimately, organ overgrowth and diseases.^290^ Studies demonstrated that *Yap* knock-out mice presented attenuation in cardiac hypertrophy and progression in fibrosis and apoptosis. This indicated that YAP could promote hypertrophy and survival in adaptive response.^291^ It was reported that YAP-regulated proliferation of small intestinal epithelial cells in the nucleus controls post-inflammatory cell regeneration and serves as a potential therapeutic target for ulcerative colitis.^292^ Under various conditions, YAP/TAZ also reprograms mature cells to a poorly differentiated state both in vivo and in vitro.^283^ Besides, YAP/TAZ also plays a crucial role in EMT,^293^ promotion of angiogenesis,^294^ hypoxic stress-induced cancer glycolysis,^295^ and metabolism.^281,296^

More importantly, YAP/TAZ responds to mechanical cues, including ECM stiffness, skeletal TF, cell geometry, cell density, substrate adhesion, and non-directional FSS, thereby regulating tumor and fibrosis progression.^149,297,298^ The currently known biological stress-promoting effects of YAP are mainly manifested in cell self-renewal,^299,300^ osteocyte differentiation,^149,301^ epithelium and vascular remodeling,^149,302,303^ epithelial damage repair,^304,305^ fibrosis,^151,306–308^ and others. Additionally, mechanical cues can modulate YAP/TAZ activity via large tumor suppressor kinase (LATS)-dependent or LATS-independent pathways. For example, it has been reported that ECM stiffness interacts with TGFβ depending on YAP/TAZ and Smad2/3. The inhibition of YAP/YAZ lowers the accumulation of Smad2/3 and alleviates renal fibrosis.^309^ NUAK family kinase1 (NUAK1) expression is triggered by YAP/TAZ to create a positive feedback loop of fibrosis.^310^ Fibroblast activation, a key result of fibrosis, is mediated by CD44/RhoA/YAP signaling under mechanical stimulation in crystalline silica-induced silicosis.^311^ ECM deposition and smooth muscle actin expression are initiated, associated with fibroblast generation and transformation.^312^ YAP has been identified to respond to ECM stiffness-induced bladder smooth muscle proliferation in urinary bladder fibrosis. Prolonged high ECM stiffness results in YAP shuttle to the nucleus and bound to Smad3 to trigger a downstream proliferative cascade.^313^ Moreover, the inhibition of mechanotransduction by YAP ablation restricts the activation of Engrailed-1 to promote wound healing mediated by Engrailed-1-negative fibroblast without scarring.^314^ Scarring is attenuated by the limitation of ECM/YAP/integrin-triggered pro-fibroblast phenotype, induced by P21-activated kinase (PAK) protein inhibition.^315^ The potential YAP/TAZ-targeted drugs have been ubiquitously investigated in several clinical trials (Table 3).

**Table 3:** Typical clinical trials targeting YAP/TAZ

Target	Intervention/treatment	Disease type	Phase	Current status	ClinicalTrials.gov identifier
YAP	Simvastatin	Prostate cancer	2	Recruiting	NCT05586360
	ION537	Advanced solid tumors	1	Completed	NCT04659096
YAP/TAZ	Zoledronate	Breast cancer	2	Terminated	NCT02347163
TEAD	IK-930	Solid tumors	1	Recruiting	NCT05228015

Besides, TRPV4 is another important mechanosensitive ion channel that responds to mechanical cues, such as ECM stiffness. For instance, ECM stiffness can induce the EMT process by activating TRPV4/AKT/YAP signaling.^316,317^ Recently, mechanical transduction has been highlighted in hearing and balance transduction. Mechanical stimulation activates transmembrane inner ear (TMIE) through direct or membrane contact with the auxiliary subunit, which binds to the transmembrane-like protein 1 (TMC-1) mechanical transduction complex, thereby opening the pore for ion permeation.^318–320^ Moreover, the six missense mutations of TMC-1 in mice all indicated a decrease in Ca^2+^ permeability.^321^ The outcomes supported that TMC-1 was the pore of the mechanoelectrical transducer channel critical in cellular mechanotransduction.

In addition, humidity receptors are also identified as mechanosensitive and thermosensitive molecules. The sensory effectors of Or42b olfactory sensory neurons are present in cuticular deformations in moist air conditions, which demonstrates the transformation from humidity into mechanical signals. Intriguingly, TMEM63 showed functional conservation in rescuing the moisture response in *Tmem63*-deficient mutant flies.^322^ Furthermore, TMEM63 is also sensitive to osmotic stress, stretching force, and negative pressure.^323,324^ However, the in-depth biochemical mechanisms of these ion channels in mechanical transduction are poorly studied, which provides novel research insights into the related fields.

## Signaling pathways in cellular mechanotransduction

Current research indicates that mechanotransduction-related diseases are associated with various signaling cascades, including TGFβ/Smad signaling pathway, Janus Kinase (JAK)/STAT signaling pathway, Wnt/β-catenin signaling pathway, ERK1/2 signaling pathway, RhoA/ROCK signaling pathway, and epigenetics signaling.^325^ Here, we elucidated several crucial signaling pathways associated with cellular mechanotransduction in normal conditions and diseases.

### RhoA/ROCK signaling pathway

RhoA belongs to the Rho-family small GTPases, which is a critical modulator of cell adhesion and cytoskeleton, and many cellular processes, such as cell migration, proliferation, and survival.^326,327^ A major downstream of RhoA is the serine-threonine kinase ROCK, which triggers actomyosin contractile force.^328,329^ The RhoA/ROCK signaling mediates cell skeleton remodeling, cell contractility, and cell death process in response to multiple biochemical and biomechanical signals.^330^

RhoA/ROCK signaling pathway is engaged in many diseases, including osteoarthritis,^331^ Alzheimer’s disease,^332^ ischemic stroke,^333^ hepatic and pulmonary fibrosis,^334,335^ and cancer.^336^ For example, the RhoA/ROCK signaling regulates the cardiac fibroblast-to-myofibroblast transformation (FMT) process.^337^ The inhibition of RhoA/ROCK attenuates the myocardial fibrosis in type II diabetes rats through JNK and TGFβ signaling pathways.^338^ The TGFβ-induced EMT process is also mediated by RhoA/ROCK signaling pathway.^339^

In response to mechanical stimulation, ECM-induced remodeling activates integrins to facilitate the RhoA/ROCK signaling pathway in cellular mechanotransduction and osteogenetic differentiation of mesenchymal stem cells.^340^ Stiff ECM-mediated integrin-induced directional migration through RhoA/ROCK signaling cascades provides new insights into inhibiting cancer metastasis.^341^ A study on mechanical stress-associated cardiac valve remodeling revealed that the mechanical stress-induced valvular fibrosis through RhoC signaling but was independent of RhoA signaling.^342^

### TGFβ/Smad signaling pathway

TGFβ/Smad is considered the canonical pathway of fibrosis.^343^ TGFβ induces the phosphorylation of Smad into the nucleus to promote downstream gene transcription. Three isoforms of TGFβ (1/2/3) have been identified. TGFβ1 and TGFβ2 are the most critical in function.^344,345^ TGFβ1 promotes the expression of transient receptor potential melastatin 7 (TRPM7) through the TGFβ/Smad3 signaling pathway and facilitates the airway smooth muscle cell proliferation.^346^

The Smad family proteins, which are the central transducers of the TGFβ signaling pathway, are classified as R-Smad, inhibitory Smad (I-Smad), and universal Smad (co-Smad, Smad4).^347^ In R-Smad, Smad2/3 are considered the main mediators of the TGFβ signaling pathway, and Smad1/5/8 mediate the BMP signaling pathway.^348^ R-Smad dissociates from phosphorylated receptors and binds to co-Smad (Smad4). This complex is a heterotrimer, with two R-Smad binding to one Smad4^349^ and one R-Smad binding to one co-Smad.^350^ Many transcription factors are reported to bind to Smad proteins.^351^ In addition, other proteins can also interact with or even modify Smad proteins. These modifications often affect the strength of signal output and interact with different signaling pathways. Smads can be modified by phosphorylation, ubiquitination, acetylation, silylation, and ADP ribosylation.^352,353^

The TGFβ/Smad signaling pathway is essential in inflammatory bone destruction,^354^ kidney disease,^355–357^ immune system,^358^ malignancy,^359^ neurological disease,^360^ and inflammatory bowel disease.^361^ Mechanical stress can activate TGFβ/Smad signaling, which drives the progression of lung fibrosis.^362^ It has been demonstrated that high mechanical stress can activate TGFβ1/connective tissue growth factor (CCN2)/integrin to induce fibrosis.^363^ TGFβ1 is modulated by cell-generated TF, promoting the disruption between TGFβ1 and latency-associated peptide (LAP).^364^ In this process, integrins interact with LAP, and integrin-mediated stretching force changes the conformation of LAP, thus triggering the release of latent TGFβ1,^365^ thereby contributing to severe fibrotic changes.

Although there are many challenges in treatment targeting TGFβ, the antagonists and monoclonal antibodies are under investigation.^366,367^ It is worth mentioning that the resolution may be targeting biochemical signaling cascades rather than TGFβ. Some studies have revealed that small molecule inhibitors can attenuate TGFβ-induced fibrosis as well.^368–371^

### JAK/STAT signaling pathway

JAK signaling pathway, also known as the interleukin (IL)-6 signaling pathway, functions in multiple biochemical processes, such as cell proliferation, cell transformation, cell apoptosis, tissue remodeling, immune regulation, and hematopoiesis.^372^ JAK is activated by binding cytokines, growth factors, or interferon to facilitate dimerization.^373^ JAK phosphorylates STAT in the cytoplasm, and the phosphorylated STAT is translocated into the nucleus to regulate gene transcriptiton.^374^

JAK/STAT pathway has been reported to be engaged in innate immunity and adaptive immune responses,^375,376^ including rheumatoid arthritis,^377^ Parkinson’s disease,^378,379^ multiple sclerosis,^380^ inflammatory bowel disease,^381^ sepsis,^382^ liver cirrhosis^383^ and tumors.^384^ In the fibrotic process, STAT3 activation triggers ECM deposition and MMPs transcriptional control.^385–387^ The interactions between TGFβ/JAK/STAT3 fibrosis, independent of Smad, have been identified.^373,388^ Besides, TGFβ can also function as a target gene of STAT. STAT3 has been reported to enhance liver fibrosis by upregulating TGFβ.^389,390^

Research from two decades ago illustrated that JAK/STAT signaling is activated by mechanical stretch in cardiomyocytes of rats, partly depending on angiotensin II.^391^ Then the JAK/STAT signaling was investigated widely, i.e., cyclical stretch triggered the expression of MMP14 and MMP2 in neonatal rat cardiomyocytes.^392^ JAK/STAT related to fibroblast activation is also identified as the downstream cascades of ROCK in mechanotransduction.^393^ TGFβ/integrin signaling cascades have been reported to be involved in the ECM alteration positive feedback process. JAK/STAT, the response of TGFβ, also functions in the cellular mechanotransduction process. Studies demonstrated that mechanical stress triggers osteogenic differentiation through JAK/STAT and PI3K/AKT signaling cascades.^394^ Mechanical stress can also communicate with intracellular signals, including JAK/STAT, signaling, and MAPK signaling, via integrins, cytoskeleton, and sarcolemmal proteins.^395^ However, some mechanisms of JAK/STAT-associated diseases in cellular mechanotransduction still need further studies.

### Wnt/β-catenin signaling pathway

As an essential downstream cascade of TGFβ-mediated fibrosis, Wnt signaling is indispensable in embryonic development and cell proliferation and migration, tumorigenesis, and cell survival through β-catenin activation.^396–398^ The Wnt proteins interact with receptor complexes on the cell surface to initiate intracellular Wnt/β-catenin signaling.^399^ β-catenin is also a transcription factor that functions through its translocation into the nucleus.

Many studies demonstrated that the Wnt/β-catenin signaling pathway involves in the fibrotic process. For instance, studies identified that the ablation of Wnt/β-catenin signaling attenuated age-associated mitochondrial dysfunction and renal fibrosis.^400^ Wnt/β-catenin signaling can facilitate wound repair of kidney injury. However, excess activation of Wnt/β-catenin in renal tubular epithelial cells and fibroblasts result in renal fibrosis.^401^ In this process, Snail1, the initial step of EMT, is activated by β-catenin in the renal tubular epithelial cells.^402^ Wnt/β-catenin can be triggered by the dynamic stimulation of ECM involved in the EMT-to-fibrosis process.^403^ In addition, the Wnt/β-catenin signaling pathway activates IL-4 and TGF-induced macrophage cell (M2) polarization to promote renal fibrosis.^404^ The activation of Wnt/β-catenin cascades induces fibroblast proliferation and differentiation to myofibroblast.^405^ Further studies revealed that the TGFβ/Smad3 signaling pathway also interacts with the Wnt/β-catenin signaling pathway in vascular remodeling.^406^

Mechanistically, Wnt/β-catenin signaling is a critical effector in mechanical transduction, including osteogenesis, cardiovascular disease, and osteoarthritis.^407–410^ Wnt/β-catenin cascades are associated with YAP/TAZ, which are important effectors of mechanical cues. Besides, Wnt/β-catenin signaling is also regulated by integrins. Wnt/β-catenin signaling was initially identified in loading-triggered osteogenesis.^411^ Osteocytes and osteoblasts respond to SS, TF, and mechanical load by triggering canonical Wnt/β-catenin cascades.^412^ Compressive mechanical stress attenuates Wnt signaling, impairing chondrocyte proliferation and cartilage matrix degradation.^413^ Studies demonstrated that β-catenin stabilization responds to tension or ECM stiffness to activate cell adhesion and E-cadherin-dependent mechanism.^414–416^ ECM stiffness also promotes EMT-induced aortic valve fibrosis through the activation of β-catenin.^417^

### ERK1/2 signaling pathway

ERK signaling pathway activates ERK1/2 phosphorylation to promote the transcription of genes.^418^ ERK1 and ERK2 belong to a family of structurally related kinases, which are also known as MAPKs. In general, the ERK signaling pathway functions in cell proliferation, cell survival, cell growth, cell metabolism, cell motility, cell differentiation, and cell development.^419^ For instance, RAS-induced ERK cascades modulate the G1/S phase transition in proliferative cells. The ablation of *Erk1/2* in mouse embryonic fibroblast triggers cell cycle arrest in the G1 phase.^420,421^ ERK1/2 can also suppress the proapoptotic factors to maintain the cell survival.^422^ Studies revealed that RAS-ERK signaling was partly engaged in Myc-induced cell growth and cell size control.^423^ ERK can phosphorylate the transcription factor HIF1α. Besides, HIF1α regulates the process of glucose metabolism.^424–426^ ERK signaling pathway activation triggers the mobility program in response to mechanical stimulation.^427^ Studies also demonstrated that ERK signaling enhancement promotes the differentiation of mouse embryonic stem cells.^428^

Furthermore, the ERK1/2 signaling pathway is closely related to fibro-proliferative disorders. The inhibition of ERK alleviates pathological vascular remodeling^429^ and biological stress regulates the proliferation of bladder smooth muscle cells through β-receptor regulation of the cAMP (EPAC)-ERK1/2 signaling pathway.^430^ In the airway remodeling of asthma model mice, IL-13 induces airway smooth muscle proliferation and hypertrophy through the ERK1/2 signaling pathway^431^; and pulmonary hypertension regulates the pulmonary artery smooth muscle proliferation through the p38MAPK/ERK signaling pathway.^432^ Rap1 regulates vascular smooth muscle proliferation through the ERK1/2 pathway.^433^ In addition, IL-11 has been reported to respond to TGFβ stimulation. In mice, fibrogenesis protein synthesis depends on the activation of IL-11 and its receptor, which leads to multiorgan fibrosis.^434^ Cytokines such as IL-9, IL-11, and IL-17A can provide novel therapeutic targets for fibrosis prevention.^435–437^

Importantly, ERK1 and ERK2 are dominant features in mechanical cues-induced diseases. The ERK signaling functions independently or cooperatively under the regulation of mechanical stress. For example, the fibrotic scar on the skin of mice, which is induced by mechanical strain, promotes the expression of leucine-rich-α-glycoprotein 1 (LRG-1). ERK inhibition attenuates the expression of LRG-1 to alleviate the scarring.^429^ The pathologic scar induced by mechanical stress is regulated by FAK/ERK pathways.^438^

### Other signaling pathways for mechanical cue-related diseases

Epigenetic regulation in various cell types functions extensively in fibrogenetic process.^439^ Epigenetics includes DNA methylation, histone modification, ncRNAs, miRNAs, RNA modification, and chromatin remodeling, which contributes to tumorigenesis, aging, and other diseases.^440–446^ In epigenetics-induced fibrosis, JMJD1 has been identified as an ECM stiffness-dependent factor regulating the transcription of YAP/TAZ and fibroblast activation.^447^ Epigenetic silencing is crucial for ECM stiffness-induced fibroblast activation.^448^

Hedgehog (Hh) signaling is a mediator for fibroblast transformation in fibrosis and consists of a series of signaling pathways in organ development, homeostasis, fibrosis, and regeneration.^449–453^ Hh signaling can be activated during macrophage cell activation and other biological responses.^454–456^ A recent study identified mechanical stress/Hh as a novel signaling cascade in fibrotic disease.^457^ Targeting Hh signaling can be a sight for the treatment of mechanical cues-induced diseases.

Hippo/YAP pathway is associated with tissue and organ development, remodeling, and several diseases. Angiotensin A2 activates YAP/TAZ, the critical downstream target of Hippo signaling, and promotes the migration and proliferation of vascular smooth muscle cells.^302^ YAP also interacts with cardiac proteins to regulate vascular remodeling.^458^ As a mechanosensitive signaling pathway, different mechanical stimulation can modulate the proliferation and apoptosis of vascular smooth muscle cells through Hippo/YAP signaling.^459^

There is evidence showing that hypoxia,^460^ Rho/ROCK signaling,^342,461^ PI3K/AKT signaling pathway,^462^ NF-κB signaling pathway,^463^ STAT3/Pim-1/NDAT signaling pathway,^464^ NLRP3 inflammasome-dependent pathway,^465^ and Notch signaling pathway^466^ are all related to the mechanical force-induced pathophysiological processes. Collectively, biomechanical signals have been extensively studied in health and diseases, but many unknown mechanisms remain to be explored.

## Cellular mechanotransduction in tissue development and repair

Mechanical stimulation is engaged in many biological activities, such as tissue development, regeneration, and remodeling. The mechanisms of cellular mechanotransduction are well-studied in skin repair, nerve regeneration, angiogenesis, vascular remodeling, and others, all of which are important in maintaining the physiological homeostasis of the human body.

### Mechanotransduction in embryonic development

Mechanotransduction in embryonic development plays a vital role in cell shape response by morphogenetic movements of tissue.^467^ Embryonic morphogenesis is closely associated with biomechanical signals upon cells and/or tissues that lead to morphological changes.^468^

ECM stiffness, FSS, and TF are principal mechanical cues in embryonic development.^469,470^ For example, in cardiovascular development, the embryonic vasculature system can sense the mechanical stimulation in vascular remodeling and certain aspects of sprouting angiogenesis.^471^ Furthermore, the mechanical properties of the embryonic heart are soon shaped from a tube that assists the nascent vasculature in the early embryogenesis process.^472^ In cardiac maturation, the increase of ECM is deposited by proliferating fibroblasts along with more sarcomeric proteins expressed by cardiomyocytes so as to increase the contractility of the heart. In addition, ECM stiffness can regulate short-term and long-term deformations of embryonic tissues through integrin-mediated adhesion and cell-cell interaction.^473^ Mechanical cues are also involved in nervous system development. FSS and HP participate in the development of cerebral ventricles. The tissue stiffness also regulates neural tube closure, neural progenitor proliferation and differentiation, and neural crest cell migration in embryonic development.^474^ Besides, embryonic tendon development is also closely correlated with tissue stiffness and dynamic mechanical loading.^475^

### Mechanotransduction in skin repair

As the largest organ in the human body, the skin maintains a strong and pliable physical structure for integrity and flexibility. Skin is mechanosensitive in human body,^476^ and multiple biomechanical properties regulate skin repair process through mechanosensitive signaling.^477^ During skin natural repair and surgery repair, skin soft tissue expansion encounters many unsolved problems, such as skin quality, tissue retraction rate, and long-term treatment.^478^

ECM is abundant in dermis, especially collagens, which provide sufficient TF in the skin. The skin TF on the actin filaments and intermediate filaments integrates integrin-mediated pathways to trigger the maturation of focal adhesion. In addition, the long-term TF facilitates papillary fibroblast proliferation, ECM deposition, and TGFβ expression, thereby promoting a satisfactory regeneration of the skin. For example, the tension within the epidermis promotes gene expression through DNA methyltransferase 3A (DNMT3A) nuclear translocation,^479^ which is a potential regulator of skin repair. However, the persistent excessive TF triggers premature papillary fibroblast, leading to poor regeneration of the skin.^480^ Many studies show that the stiffness of skin tissue ensures the regenerative ability during the wound healing process. The ECM secreted and remodeled by matrix metalloproteases realizes a complex balance for the dermis in tissue injury.^481^ Of note, the skin repair process is always followed by hypertrophic scar formation, which is characterized by skin proliferative disease. For example, fibroblast is highly mechanosensitive, which is responsible for scar formation.^482^ Piezo1 is highly expressed in hypertrophic scar,^483^ so targeting mechanosensitive effectors may be a potential therapeutic targets for the scar.

### Mechanotransduction in nervous system regeneration

Recent studies have been focusing on the impact of biomechanical cues on the nervous system. Mechanical cues are engaged in neuron development, proliferation, differentiation, migration, and axon extension. Multiple mechanics-associated signaling pathways (i.e., Hippo signaling pathway) are essential for nervous system development and nerve function maintenance.^484,485^ For example, YAP/TAZ can integrate biochemical and biomechanical signals to promote myelination via the activation of Schwann cells proliferation and transcription of basal lamina receptor genes, both of which are essential for myelination. ECM stiffness-induced YAP/TAZ activation triggers the axon outgrowth by actin-adhesion coupling.^486,487^ A recent study demonstrated that mechanosensitive ion channels, such as Piezo1 and Piezo2, are rich in Schwann cells. Piezo1 is a transient inhibitor of radial and longitudinal myelination, but Piezo2 may be essential for myelination in Schwann cells.^488^ Researchers also found that human motor neurons tended to survive in rigid conditions with the elasticity of muscle, but human forebrain neurons preferred brain tissue-like elasticity.^489^

For neural development, the embryonic cerebrospinal fluid (CSF) is secreted into the lumen of the brain and ventricles. The CSF pressure triggers a dramatic dynamic expansion of the brain.^490^ The telencephalon developing to left and right lobes are associated with CSF-induced mechanical environment.^491^ The central nervous system is mechanically induced only by tangential growth but not radial growth. This suggests that the mechanosensors and transducers are different in the nervous system.^492,493^

Apart from endogenous mechanical stimulation, TF can also promote axon growth.^494,495^ Cell motility and adhesion are both involved in the determination of the extension of axon and growth cone activities.^496,497^ In general, axonal transport is driven by mechanical methods with the help of several motor proteins such as myosin, kinesin, and dynesin.^498^ Hence, more studies of mechanical cues-regulated mechanisms are to be conducted for the nervous system.

### Mechanotransduction in angiogenesis and vascular remodeling

Angiogenesis is a multi-stage process characterized by endothelial cells activated by angiogenin, VEGF, platelet-derived growth factor (PDGF), epidermal growth factor (EGF), fibroblast growth factor, TGFβ, and tumor necrosis factor α (TNFα).^499^ For morphological changes in endothelial cells, pericytes detach from endothelial cells that release metalloproteases to degrade ECM.^500^ Vascular tubes are formed by tip cell migration and stalk cells proliferation.^501^ The mechanisms of angiogenesis above also meet the requirements in embryonic development.

Mechanical cues, such as ECM stiffness, TF, and FSS, play important roles in angiogenesis and vascular remodeling. Stiffing ECM induces pro-angiogenesis gene expression via YAP activation, such as *Vegfa*, *Vegfb*,^502^ thereby promoting angiogenesis. In addition, TF in blood vessels generated by blood circulation activates mechanosensitive proteins (i.e., Piezo1 and TRPV4) and facilitates biomechanical signaling pathways. For instance, the activation of Piezo1 upregulates membrane type1 MMP, AKT, and mTOR signaling,^503,504^ thereby increasing VEGF expression. Besides, FSS in blood vessels remodels endothelial cells by activating Piezo1, TRPV4, tyrosine kinase receptors (TKRs), G-protein-coupled receptors, and integrins.^505,506^ Branches and bends of arteries have intricate blood flow patterns that can trigger vascular dysfunction. However, physiologically high FSS is protective for angiogenesis and vascular remodeling by regulating BMP-TGFβ, WNT, Notch, hypoxia-inducible factor 1α (HIF1α), TWIST1, and HOX family genes.^507^ A previous study showed that higher FSS reduced vascular density in veins but not in arteries during vascular development.^508^

Mechanical strain and FSS in blood vessels are the main mechanical cues in vascular remodeling. In several disease progression stages, the increased FSS induces vascular remodeling to maintain enough tissue perfusion. VEGFR2, VEGFR3, and VE-cadherin communicate with transmembrane domains (TMDs) within the plasma membrane. FSS activates PECAM-1, leading to the activation of Src and VEGFRs.^509^ In the vascular remodeling process, ROS, NO, NF-κB, epidermal growth factor receptor (EGFR), MAPK, and PKC signaling pathways are all activated by mechanical cues.^510,511^ For (myo)fibroblasts, mechanical stretch can significantly increase the expression of β1 integrin, α-SMA, Nesprin2, and laminA/C. TF modulates the assembly of α-SMA through the Rho kinase pathway^512^ and the fibroblasts are transformed into α-SMA^+^ myofibroblasts. Other studies have found that the TF can promote fibroblast differentiation by activating TGFβ1, thereby promoting the remodeling of tissues and organs.^103^ Cyclic stretching can also rearrange microfilaments in human periodontal ligament (PDL) cells, thereby regulating cytoskeleton-related changes of gene expression.^513^ This process may be affected by the Rho signaling pathway,^514^ but the mechanism is still unknown.

## Mechanotransduction in diseases

Although the normal range of physiological mechanical stimulation exhibits a critical impact on the sustainment of cell function, it is important to note that continuous excessive mechanical stimulation is associated with a wide range of diseases and pathologic conditions, including tissue injury and fibrosis, tumor behaviors, cardiovascular diseases, and aging.

### Tissue injury and fibrosis

Fibrosis is an excessive tissue repair process induced by multiple tissue damage factors. The combined annualized incidence is approximately 4968 out of 100,000 person-years globally.^515^ Although the fibrotic response may only cause minor changes to tissues in the early stage, parenchymal sclerosis and cell dysfunction may occur long after. The fibrotic responses generally consist of the primary organ injury stage, effector cells (fibroblasts, myofibroblasts, bone marrow, fibrocytes, epithelial-derived cells in EMT) activation stage, ECM deposition, and loss of cellular homeostasis, which ultimately leads to organ failure.^325,516^ When the tissue receives a single or minor injury, the tissue repair process will be activated to rebuild the tissue structure. If the tissue suffers repeated or severe damage, the ECM will excessively deposit, eventually leading to fibrosis.

#### Etiology and mechanism of fibrosis

Fibrosis is often the sign of the final disease stage and is not easily reversed.^517^ The etiologies of fibrosis include persistent infections, toxin exposures, inherited disorders, chronic autoimmune inflammations, myocardial diseases, abnormal serum cholesterol, obesity, smoking, severe diabetes, and blood hypertension.^518^ Many organ disorders can induce fibrotic diseases, such as liver cirrhosis, kidney fibrosis, bladder outlet obstruction, IPF, cardiac fibrosis, corneal trauma, glaucoma, atherosclerosis, scleroderma, skin disorders, burns, radiation-induced fibrosis, chemotherapy-induced fibrosis, and surgical complications.^116,429,477,519,520^ Besides, chronic autoimmune diseases are also identified as potential causes of fibrosis, including rheumatoid arthritis, scleroderma, inflammatory bowel disease, myelosclerosis, and systemic lupus erythematosus.^521–523^ Since fibrosis causes irreversible damage to organs, identifying the exact mechanisms would provide effective therapies for preventing fibrosis.

**Fig. 6:**
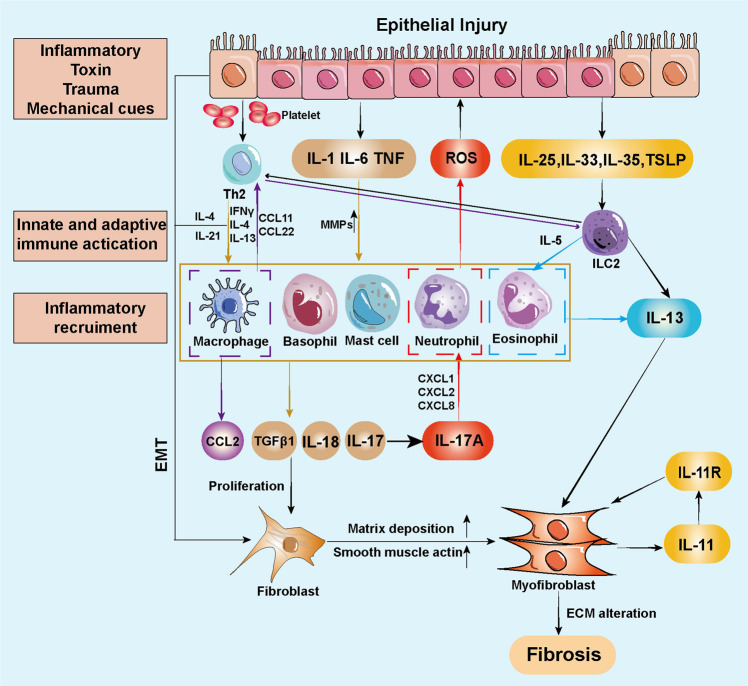
A schematic view of the fibrotic response. The innate and adaptive immune systems are activated at the tissue repair stage. The pro-inflammatory chemokines and cytokines secreted by macrophage cells trigger the inflammatory recruitment process. TGFβ1, IL-17, and IL-18 from immune cells promote fibroblast proliferation and transformation. In addition, type I and type II immunity are cross-activated for defense and metabolic homeostasis maintenance. The adaptive immune CD4^+^ Th2 cell can directly activate fibroblasts by IL-4 and IL-21, independent of TGFβ1 induced fibrosis. Excessive ECM deposition leads to fibrosis ultimately. ECM extracellular matrix, EMT epithelial-to-mesenchymal transition, IL interleukin, ILC2 group 2 innate lymphoid cells, MMP matrix metalloprotease, Th2 T helper 2, TGFβ1 transforming growth factor β1, TNF tumor necrosis factor

During fibrosis, myofibroblasts have long been recognized as core cell components in remodeling tissue. Therefore, FMT is critical in ECM accumulation and scarring processes. Multiple studies have shown that fibrosis triggers (myo)fibroblasts,^524^ muscle cells,^525^ and also recruits inflammatory cells (i.e., macrophages and T cells).^526^ When the tissue suffers an injury, the innate and adaptive immune responses will be activated^527^ (Fig. 6). Type I and Type II immunity are cross-activated for defense and metabolic homeostasis maintenance. Type II immunity involves cytokines (i.e., IL-4, IL-5, IL-9, IL-13, IL-25, and IL-33), thymic stromal lymphopoietin, eosinophils, basophils, mast cells, T helper 2 (Th2) cells, group 2 innate lymphoid cells (ILC2s), and IL-4/13-activated macrophages.^528–531^ The pro-inflammatory chemokines and cytokines (i.e., IL-1, IL-6, and TNF) secreted by macrophage cells, trigger the inflammatory recruitment process.^532^ TGFβ1, IL-17, and IL-18 can promote fibroblast proliferation as well.^533^ Meanwhile, IL-17A strengthens the neutrophil responses by CXCL1/2/8 and aggravates tissue injury by synthesizing reactive oxygen species (ROS).^534^ The IL-17A signaling increases the TGFβ1 receptors on fibroblasts and facilitates the secretion of ECM. The adaptive immune CD4^+^ Th2 cell can directly activate fibroblasts via IL-4 and IL-21, independent of TGFβ1-induced fibrosis.^535,536^ Intriguingly, it has been demonstrated that IL-4 is a potent profibrotic cytokine that is more effective than TGFβ.^537,538^ Similarly, LC2s-originated IL-5 can recruit and activate eosinophils. IL-5 has been identified in attenuating liver cirrhosis^539^ and intestinal fibrosis^540^ in mice. Eosinophils secrete IL-13 and TGFβ1 and both can facilitate the fibrotic function of the myofibroblast. Besides, IL-13 acts together with IL-4 to promote fibroblast proliferation and FMT.^541^ However, the efficacy of anti-IL-13 therapy in ulcerative colitis is controversial,^542^ and further investigations are required to identify the effect of IL-13 in the fibrotic alterations of Crohn’s disease.^543,544^ Fibroblasts can be activated directly by etiologies such as inflammations, toxins, mechanical force, and trauma through EMT process.^545–547^ However, renal fibrosis is only alleviated when IL-33/ILC2 is pre-inhibited in the unilateral ureteral obstruction model, while post-inhibitions tend to be useless.^548^

**Fig. 7:**
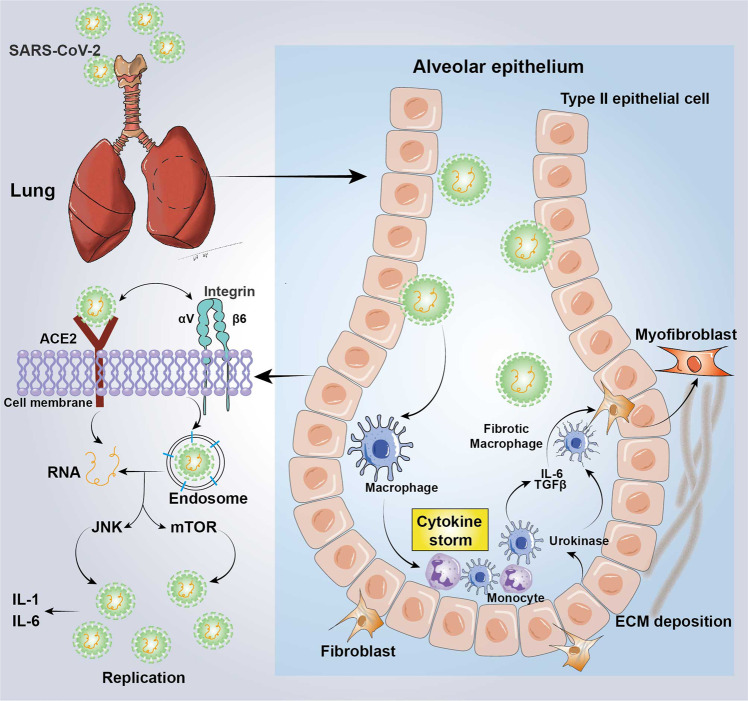
Biochemical mechanisms of SARS-CoV-2-induced lung fibrosis. SARS-CoV-2 initially binds to ACE2 of the epithelial cells to activate integrins or CD98. Integrins, especially αVβ6, can assist the SARS-CoV-2 binding to ACE2, thus enhancing the ability of viral infectivity. After SARS-CoV-2 is inhaled, the virus replicates through the JNK and mTOR signaling, which facilitate the generation of NLRP3 inflammasome. Meanwhile, the type II alveolar epithelium cell injury induces pro-inflammatory recruitment of immune cells, such as macrophages. The cytokine storm will then be released and triggers the proliferation and migration of fibroblasts. Besides, the activation of TGFβ triggered by integrins, IL-1, and IL-6 promotes ECM deposition and FMT. ACE2 angiotensin-converting enzyme 2, CAMK calmodulin kinase, SARS-CoV-2 severe acute respiratory syndrome coronavirus 2, ECM extracellular matrix, FMT fibroblast-to-myofibroblast transformation, IL interleukin, TGFβ1 transforming growth factor β1

Apart from the common etiologies, the newly emerged coronavirus disease 2019 (COVID-19) pandemic, caused by severe acute respiratory syndrome coronavirus 2 (SARS-CoV-2), can also lead to pulmonary fibrosis (Fig. 7). COVID-19 is manifested as a severe respiratory syndrome and leads to a worldwide pandemic.^549^ To date (Apr.5, 2023), there are already more than 761 million cases of COVID-19 occurrence and more than 6.89 million deaths from COVID-19 worldwide. An abrupt increase in transmission has arisen because of the Omicron variant (Nov. 26, 2021, WHO).^550^ The main symptoms of COVID-19 are generally asymptomatic infections, such as sore throat, headache, and fatigue.^551^ In some cases, the conditions progress to severe pulmonary infections requiring intensive care or even mechanical ventilation (MV).^552^ It is worth noticing that even though COVID-19-related fibrosis was observed to be relieved in one-third of the patients after four months from onset,^553^ most post-COVID-19 fibrotic lesions still cannot be cured.

#### Overview of mechanical cues-induced fibrosis

ECM deposition destabilizes the tissue’s mechanical environment,^554^ along with the change of cell survival.^555^ The expansion and contraction of hollow organs and the fluid flow in the cavities produce mechanical stimulation on tissues and organs, such as HP, FSS, and TF.^556–559^ In addition, ECM deposition can exacerbate intrastromal cross-linking, increase tension of the tissues, and induce fibrosis.^118^ These common mechanical cues function as a double-edged sword: physiological mechanical signals ensure the normal development and repair of organs, while prolonged pathological mechanical stimulation causes irreversible damage.

Fibrosis-induced alteration of ECM environments promotes abnormal biological processes. In adipose tissue, ECM-associated mechanical cues do not induce regional apoptosis and inflammation, but trigger system-wide lipotoxicity and insulin resistance.^560^ Both integrins and Piezo channels are located in the cell membrane, which can sense the mechanical cues in regulating different biological processes. The integrins mediate biomechanical signals to affect cell adhesion, migration, proliferation, and differentiation.^561,562^ For example, MV can aggravate sepsis-related pulmonary fibrosis, where MV leads to lung fibroblast proliferation, ECM deposition in lung tissue, and increase in procollagen type I carboxy-terminal propeptide in the bronchoalveolar lavage fluid mediated by β3 integrin.^563^ Another hypothesis demonstrated that MV accelerates acute respiratory distress syndrome (ARDS)-associated pulmonary fibrosis via Piezo1-mediated Ca^2+^ influx and ATP release in lung epithelial cells.^564^ In addition, physiological stress promotes the development and structural stability of tissues and organs, while pathological stress disrupts this balance, thus resulting in irreversible damage to anatomical functions.^565^

Mechanical cues can target nearly all types of cells in the human body. Fibroblast, a common functional cell type in fibrosis, responds to the mechanical cues in fibrosis progression. In the early stage of tissue injury, mechanical cues integrates with inflammatory factors to enhance tissue adhesion and fibrosis.^566^ For example, the elevated ECM stiffness regulates fibroblast DNA methylation and chromatin condensation.^567^ Persistent fibroblast phenotype forms after the first week’s rapid response to ECM stiffness. In myocardial fibrosis, *Selenbp1* knockdown enhances fibroblast activation and deters fibroblast transformation to matrix-degrading form.^568^ Mechanical cues activate TGFβ1, which is latent in ECM, subsequently activating fibroblasts.^569^ The activated fibroblasts are transformed to injury-related fibroblasts subtype, promoting ECM secretion. In addition, epithelial and endothelial cells can also sense changes in intracellular mechanical signals during tissue damage. Mechanosensors of epithelial and endothelial cells include adhesion protein complexes, primary cilia, and mechanosensitive ion channels.^199,570^ The activation of sensors can lead to functional alterations of the endothelial and epithelial barrier, inflammatory signaling, arteriogenesis, cell migration, invasion, adhesion, and proliferation.^61,571^ Endothelial cells transduce signals to the surrounding microenvironment through growth factors and chemokines.^572^ ECM can activate the cell status and promote fibrosis progression through biomechanical signals in a continuous and persistent manner.^573^ Apart from epithelium and endothelium, smooth muscle cells, HSCs, and renal tubular cells can also serves in cellular mechanotransduction.

Furthermore, several mechanical cues-induced signaling pathways are engaged in muscarinic receptor-mediated fibrosis. Muscarinic receptor-mediated HP/NF-κB induces the pro-inflammatory processes in the urothelium and smooth muscle of the urinary bladder.^25,574^ Similar performances are also observed in β adrenoceptors and integrin/FAK signaling.^430,575,576^ However, the β2 adrenoceptors in the heart can strengthen heart contraction and heartbeat by coupling with adenylyl cyclase, which is contrary to the observations in the urinary bladder.^577,578^ It is speculated that tissue heterogeneity is a possible reason for this and further validations are required. In addition, the monocyte chemotactic protein 1 (MCP1) and its receptor chemokine (C-C motif) receptor 2 (CCR2), and MCP1-induced protein (MCPIP1) are also engaged in mechanical cues-induced fibrosis. Pathological HP regulates the human bladder smooth muscle cell proliferation through MCP1/CCR2-SGK-1 signaling and contraction through MCP1/CCR2-MCPIP1.^579,580^ Therefore, HP is important in tissue repair and reconstruction, also in the exploration of therapeutic targets for fibrosis.

#### Mechanotransduction in pulmonary fibrosis

IPF is an interstitial pulmonary disease affecting gas exchange, and eventually leads to respiratory failure.^581^ Globally, the annual prevalence of IPF increases annually, with 8.65 per 100,000 per year in the United Kingdom (UK) from 2000 to 2012, and 1.2–4.6 per 100,000 per year in East Asia.^582^ Pulmonary fibrosis is evaluated by pulmonary functional testing, such as forced vital capacity (FVC), which reflects respiratory compliance.^583^ To date, the etiologies and mechanisms of IPF have not been clarified and no effective treatments have been found.

The mechanical cues in the respiratory cycle include air stress and tissue strain. These two mechanical cues act on ECM to facilitate the crosstalk between the matrix and the cell. In the lung, the ECM compartments, including basement membranes, are composed of glycoproteins, located on the epithelium and endothelium, and interstitial matrices, with loose and fibril-like meshwork with cross-linking structures and mesenchymal cells to maintain mechanical homeostasis.^584^ Persistent excessive stress and strain may cause abnormal deposition of ECM, leading to changes in ECM stiffness. In the lung, Young’s modulus of normal tissue is approximately 1.8 kPa (range ~0.2–3.7 kPa) while in pulmonary fibrotic tissue approximately 15.5 kPa (range ~3.6–54.1 kPa).^585^

All cells in the lung are regulated by mechanical stimulation. In general, the characteristics of IPF are epithelial cells aging and ECM deposition. In the normal tissue repair process, myofibroblasts are activated to secret ECM so as to facilitate AEC2 progenitor proliferation and differentiation, and thus repairing epithelium.^586^ However, a failed repair of alveolar epithelium repair may cause aberrant mesenchymal activation. Meanwhile, innate immune cells, such as monocyte-derived alveolar macrophages are important for pulmonary fibrosis.^587^ Mechanical stress can induce mast cell degranulation to activate TGFβ signaling in pulmonary fibrosis progression.^588^

For signaling transduction of pulmonary fibrosis, tyrosine kinase, and serine-threonine kinase pathways, G-protein-coupled pathways are associated with myofibroblast differentiation, matrix deposition, fibroblast proliferation, and reduced apoptosis. ECM stiffness and mechanical signaling (i.e., RhoA/ROCK, myocardin-related transcription factor-A [MRTF-A], and YAP/TAZ) promote pulmonary fibrosis via TRPV4-related signaling and others. Besides ECM stiffness, insufficient tissue repair leads to ECM component alteration, resulting in a mechanically stretched niche. The MV facilitates the TF to the lung and initiates acute respiratory distress syndrome (ARDS)-related pulmonary fibrosis. Mechanical stretch impairs the tight junction between alveolar cells. The loss of tight junction leads to the degradation of the cytoskeleton and cell-cell attachment. Tang et al. demonstrated that an elevated mechanical TF activates the TGFβ signaling loop in alveolar stem (AT2) cells.^362^ Tang et al. found that AT2 cells in lungs distribute less uniformly under high pressure than under low pressure. Moreover, a recent study demonstrated that mechanical stress induces midkine (MK), a novel cytokine, to promote the pathogenesis of pulmonary fibrosis, which is a potential therapeutic target in mechanical stress-induced fibrosis.^589^

Generally, pulmonary fibrosis initiates from the lung periphery. According to the pathological features of pulmonary fibrosis, researchers hypothesized that the peripheral region of the lung withstand higher TF, thereby increasing ECM stiffness and contributing to pulmonary fibrosis progression and lung cancer.^590^ Mechanistically, YAP responds to TF to activate mTOR/PI3K/AKT signaling to regulate epithelial cell proliferation, differentiation, and migration.^591^ In addition, ECM components and stiffness are also important regulators for pulmonary fibrosis. Cross-linking enzymes, including LOX, LOXL, and transglutaminases (TGs), enhance fibroblast accumulation and increase resistance to ECM.^592^ Meanwhile, TF from alveolar epithelial cells induces ECM deposition, leading to an increase in ECM stiffness. Increased ECM stiffness and TF activate TGFβ and integrin to promote the activation of fibroblasts and epithelial cells in the lung tissue. For example, the scarring tissue in pulmonary fibrosis is from excessive deposition of ECM. The elevated ECM stiffness enhances tank binding protein kinase 1 (TBK1) phosphorylation to activate YAP/TAZ, which is independent of the Hippo signaling pathway. In addition, mouse minute 4 homolog (MDM4) is identified as an ECM stiffness-modulated endogenous inhibitor of p53.^593^ Targeting MDM4-p53 (myo)fibroblast can help reduce pulmonary matrix through nonenzymatic cross-linking or genetic ablation. Interestingly, fibroblast can also be activated by CD44/RhoA/YAP-mediated mechanics-induced pulmonary fibrosis.^311^ Recent research demonstrated that ANKED42, a novel circRNA-ankyrin repeat domain 42, sponges miR324-5p to activate AJUBA expression, inhibiting the interaction between phosphorylated YAP and LATS1/2, thus resulting in YAP translocation to the nucleus. Eventually, pulmonary fibrosis is strengthened by mechanical cues-mediated YAP activation.^594^

#### Mechanotransduction in liver fibrosis

Liver fibrosis is a pathological process induced by multiple factors and eventually leads to liver dysfunction. The etiologies of liver fibrosis include viral hepatitis B/C, autoimmune liver diseases, non-alcoholic fatty liver disease (NAFLD), hereditary diseases (i.e., Wilson’s disease), and liver cancer.^595^ Portal hypertension (PH) and ECM stiffness are of the two main mechanical cues in liver fibrosis progression. PH is categorized by presinusoidal, sinusoidal, and post-sinusoidal.^596^ Sinusoidal compression increase and vascular compliance reduction can lead to elevated portal pressure. In the HP-induced liver fibrosis model, losartan could alleviates mechanotransduction-associated fibrosis in HSCs.^21^ As a result of continuous abnormal PH stimulation, elevated ECM deposition increases the stiffness of the matrix, which is also a promoter of liver fibrosis. Losartan has already been applied as the therapy for hepatitis C with mild liver fibrosis (NCT00298714) and non-alcoholic steatohepatitis (NCT01051219).

HSCs, hepatocytes, portal fibroblasts, and immunocytes are the main cell types activated during liver fibrosis. Various studies showed different behaviors of hepatocytes on soft supports and dedifferentiated behaviors on stiff supports in liver fibrosis progression. ECM stiffness has also been reported to influence the hepatocytes’ response to growth factors and fibronectin density.^597^ HSCs are typical mesenchymal cells with the characteristics of both fibroblasts and pericytes. As a major precursor of the myofibroblasts in the liver, quiescent HSCs can be activated by mechanical cues, such as ECM stiffness. The mechanical stimulation transforms HSCs to myofibroblasts.^598^

Meanwhile, the features of cell proliferation, cell migration, and cell contraction are facilitated by mechanical cues, leading to high secretion of ECM macromolecule. During ECM deposition in the space of Disse, the ECM composition changes from collagen type IV, heparan sulfate proteoglycan, and lamin to fibrillar collagen type I and III.^599^ The integrinαv, cytoskeleton-dependent actin-myosin, and Rho/GTPases can respond to mechanical stimulations in the liver.^263,600^ Moreover, ECM stiffness also activates myofibroblastic differentiation in vivo. Both ECM stiffness and TGFβ trigger portal fibroblast activation in biliary fibrosis.^601^ A recent study showed that ECM stiffness-induced plasma membrane tension can be orchestrated by the integrinβ1/RhoA axis, which promotes the expression of the tissue inhibitors, metalloproteinase 1 (TIMP-1) and caveolin-1, in liver cirrhosis.^602^ In addition, increased ECM also binds to, hepatocyte growth factor (HGF), fibroblast growth factor (FGF), epidermal growth factor (EGF), and VEGF, facilitating HSC proliferation.^603^ The crosslinking enzyme lysyl oxidase-like-2 (LOXL2), mainly expressed by HSCs, catalyzes collagens and elastins crosslinking.^604,605^ LOXL2 is regarded as a therapeutic target in the simtuzumab (GS-6624) testing of phase II clinical trials, such as NCT01707472, NCT01672853, NCT01672866, and NCT01672879.

#### Mechanotransduction in renal fibrosis

Renal fibrosis is generally caused by renal tubule injury and may result in glomerulosclerosis, tubulointerstitial fibrosis, and angiosclerosis.^606^ One of the features of renal fibrosis is tissue scarring caused by excessive ECM deposition, which is a common outcome of multiple chronic kidney diseases.^607^

The kidney cells are living in a blood-shear environment sheltered by the nephron. FSS in the nephron ranges from 0.06 to 0.3 dyn/cm^2^.^608,609^ FSS is a typical mechanical cue being discussed in many fibrotic processes. Essig et al. found that FSS (0.17 dyn/cm^2^) regulates the fibrotic process through decreasing the expression of plasminogen activator and urokinase.^609^ Moreover, the TGFβ-induced EMT of renal epithelial cells is modulated by mechanical responses, in which process the stretching of renal cells activates MAPK signaling.^610^

Apart from FSS, TF from urological obstruction also plays an important role in renal fibrosis. The ureteral obstruction-related hydronephrosis leads to elevated intratubular pressure.^611^ The obstruction leads to a sharp increase in fluid pressure that damages the renal tubules. Although the compensatory dilation of the pelvis can alleviate the immediate pressure increase, the pressure will still cause damage to the nephron gradually. The nephron cells sense the FSS and HP through activated ion channels, thereby promoting renal fibrosis.^612^ TF is reported to activate TGFβ-induced EMT as well.^613,614^ The mechanical TF upregulates fibronectin and TGFβ, activates the signal transducer and transcription factor 3 (STAT3), thereby promoting renal fibrosis.^615^ Meanwhile, excessive ROS can also be generated by the activation of TF, which results in renal injury.^616^ ROS in turn facilitates the cytoplasmic proline-rich tyrosine kinase 2 (Pyk2), which is parallel to the expression of TGFβ.

There are three main mechanisms between renal epithelial cells and mechanical cues: the torque of a large number of apical microvilli, the bending moment of primary cilia, and the activation of mechanically sensitive ion channels that activate specific signaling.^617^ It has been reported that cilia might be associated with fluid resorption in response to FSS.^618^ This indicates that primary cilia may be engaged in the mechanotransduction in renal epithelial cells. Nevertheless, superficial luminal FSS of kidney cells triggers a Ca^2+^ response without cilium.^619,620^

In general, fibrotic lesions are distributed at localized fibrogenic niches.^621^ The ECM-regulated specialized microenvironment mainly includes ECM macromolecules, resident and infiltrating inflammatory cells of the kidney, extracellular vesicles, soluble factors, and metabolites. The pathological ECM environment promotes renal fibroblast proliferation, tubular injury, macrophage activation, and endothelial cell depletion. The ECM mechanical signals can interact with integrin αv, which further activates TGFβ/Smad signaling to promote renal fibrosis.^622^ In addition, macrophages in the kidney can sense FSS and ECM stiffness to promote renal fibrosis. Mechanical cues-activated Piezo1 channel facilitates the vascular formation and blood pressure modulation to inflammatory responses. A recent study demonstrated that the depletion of *Piezo1* can inhibit macrophage inflammation, thereby alleviating renal fibrosis and EMT process.^623^ YAP is another important sensor in mechanotransduction and has long been considered a critical regulator in myofibroblast transformation. A recent study revealed that inhibition of YTHDF1 can alleviate renal fibrosis by targeting YAP. However, the effective drugs targeting renal fibrosis still remains challenging.

#### Mechanotransduction in cardiac fibrosis

Cardiac fibrosis is characterized by excessive ECM deposition in the cardiac interstitium, which impairs both systolic and diastolic functions of the cardiac system,^624^ eventually leading to heart failure.^625^ Although cardiac fibrosis is always associated with adverse outcomes of myocardial diseases, it is not the primary cause of cardiac dysfunction. Initially, cardiac fibrosis presents a reparative behavior, the formation of collagen-based scar substitutes the myocardiocytes to maintain the elasticity and prevent the infiltration of injury and cardiac rupture,^626^ such as myocardial infarction. However, fibrosis involves only the interstitium in other cardiac diseases without impairments to cardiomyocytes.

In the human heart, the cardiac ECM is a three-dimensional scaffold that defines the geometry and muscular structure of the cardiac chambers and transmits forces generated during cardiac systolation and diastolation.^627^ Generally, the cardiac matrix is divided into epimysium, perimysium and endomysium.^628^ The valve leaflets, chordae tendineae, and collagen matrix of the myocardium are connected by type I collagen and type III collagen fibers. The abnormal mechanical strength initiates the heart remodeling process, with elevated ECM stiffness, fibroblast proliferation, and differentiation, and cardiomyocytes pathological hypertrophy. For instance, blood hypertension triggers the deposition of ECM and disturbance of the mechanical environment of the heart progressively, leading to diastolic dysfunction.

The mechanical environment in the cardiac system is complex, including HP, TF, FSS, and ECM stiffness. Ventricular hypertension is an important factor contributing to cardiac remodeling and fibrosis. For example, single-cell RNA-seq (scRNA-seq), spatial transcriptomics, and genetic perturbation demonstrated that the pressure overload downregulates the expression of high-temperature requirement A serine peptidase 3 (HTRA3) in cardiac fibroblasts and triggers TGFβ-mediated signaling cascades, eventually leading to cardiac fibrosis.^629^ Similar to other mechanics-induced remodeling processes, the pressure overload activates YAP/TAZ-dependent fibrotic signaling transduction.^630^ Long-term overload pressure and stretch lead to abnormal ECM deposition in the cardiac interstitium, which results in elevated ECM stiffness. The stiff matrix imposes mechanical stimulation on myofibroblasts to facilitate cardiac fibrosis progression. TRP family proteins are sensitive to mechanical stimulation. For example, ECM stiffness facilitates myofibroblast activation and differentiation.^631^ In cardiac fibrosis progression, stiff matrix enhances the proliferation of cardiac fibroblasts by activating YAP-mediated TEA-binding domain (TEAD) and RUNX2 transcription.^632^ More importantly, the cardiac tissue scarring in fibrosis can prevent the heart from rupture. In turn, cardiac fibrosis can not be reversed, eventually leading to heart failure. Hence, recent studies suggested that cultured cardiac fibroblasts can be transformed into cardiomyocytes, which are exciting evidence for the protection of cardiac function.^633^

Fibroblasts are the main effectors of cardiac fibrosis. Cardiac fibroblast can sense mechanical cues through mechanosensitive receptors, and channels, hence promoting intracellular fibrogenic signaling.^634^ Previous studies demonstrated that cyclic stretch promotes fibroblast activation and FMT. The inhibition of TGFβ significantly deters TF-induced expression of SMA and collagens by attenuating the phosphorylation of Smad2.^635^ The cyclic strain of fibroblasts was shown to elevate ECM deposition (i.e., collagens and fibronectin) via protein kinase C and tyrosine kinase signaling.^636,637^ In addition, ERK1/2 and NF-κB signaling also respond to the cyclic strain on cardiac fibroblasts.^638,639^ Besides, atrial fibrillation (AF)-associated with abnormal atrial stretch is also a factor contributing to cardiac fibrosis. The chronic atrial overload caused by AF leads to cardiac fibroblast proliferation, apoptosis, and morphology change. The activation of the G-protein-coupled receptor triggers ERK1/2 and JNK signaling via phosphorylation of c-Jun, c-Fos, and Fra-1.^640^ Further studies are warranted to identify the mechanisms and therapeutic targets for cardiac fibrosis.

### Mechanotransduction in cancer cell behavior

Tumor microenvironment (TME) is an important factor affecting pathological changes for multiple tumors. As a type of TME, organ or tissue mechanical properties can change the cancer cell behaviors (i.e., growth, migration, invasion, metastasis, dedifferentiation) and are different in tumors and normal tissues. Hence, cancer cell mechanotransduction signaling cascades are important therapeutic targets for anti-cancer therapy. However, the mechanisms between mechanical cues and cancer cell behaviors are still not fully elucidated.

#### Mechanisms of mechanical cues-induced cancer cell behavior

**Fig. 8:**
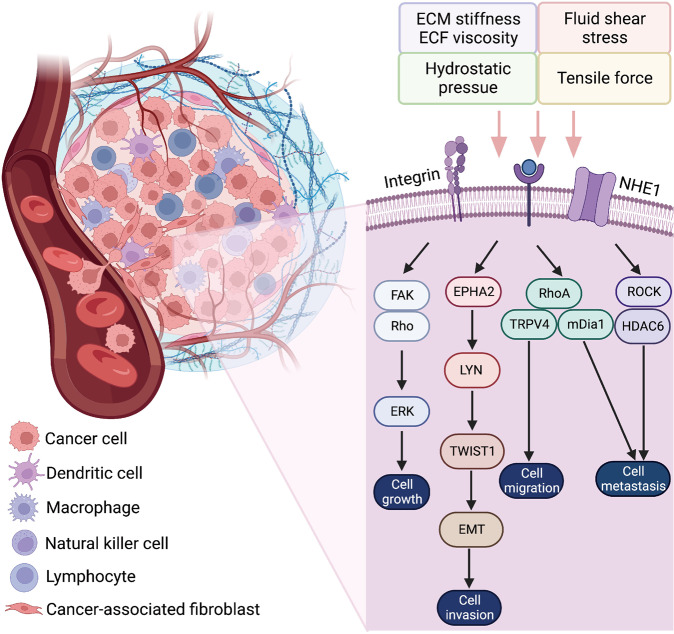
The mechanisms of mechanical cues-induced cancer cell behavior in tumor microenvironment. Mechanical cues induced cancer cell growth, invasion, migration and metastasis in multiple signaling pathways. Cancer-associated fibroblasts engage in ECM remodeling process, which trigger cancer cell migration and dissemination. ECF extracellular fluid, ECM extracellular matrix, EMT epithelial-to-mesenchymal transition, HDAC6 histone deacetylase 6, NHE1 Na+/H+ exchanger 1, TRPV4 transient receptor potential vanilloid 4. This figure was created using Biorender.com

Multiple studies have reported that ECM functions in cancer biology. ECM microenvironment serves as not only a nest for cancer and stroma cells but also a reservoir for cytokines and growth factors.^641^ Apart from the crosstalk between cancer cells and matrix, ECM stiffness is also engaged in the regulation of cancer cells, such as cell proliferation, cell differentiation, cell migration, cell invasion, EMT, and metabolic reprogramming^642,643^ (Fig. 8). ECM stiffness typically affects the movements of the lipid bilayer and the activation of ion channels. The ion channels transmit biomechanical signals to cell biochemical signaling to promote cancer cell migration.^644,645^

The elasticity of tumor tissue is higher than that of the surrounding normal tissue, which aroused focus from the researchers.^646^ For tumor growth, the elevated ECM stiffness triggers Rho-dependent cytoskeletal tension that enhances cell adhesion and disrupts cell junctions, thereby promoting cancer cell growth.^647^ ECM stiffness transmits prolactin signals to protumorigenic Src/FAK and MMP activation.^648^ Moreover, ECM stiffness could also induces epithelial cell growth via the FAK/Rho/ERK signaling pathway.^649^ In general, ECM cross-linking is regarded as the barrier preventing cancer cells from migration. However, it has been found that cancer cell migration is facilitated by elevated ECM stiffness. Cancer cell migration is initiated by CAFs, which secret protease to remodel the ECM and break through the ECM barrier.^650^ CAFs commonly express high-level α-SMA, which may be activated by TGFβ.^651^ CAFs can also be activated by mechanical cues via MRTF-SRF and YAP-TEAD signaling pathways.^652^ Together, ECM stiffness, FSS, HP, and TF are mechanical properties that trigger cancer cell behaviors.^653^

In addition, cancer cell migration from one site to another requires cytoskeleton remodeling. Stiff ECM triggers actomyosin contractility to activate RhoA/mDia1 signaling and microtubule network reconstruction, promoting cancer metastasis.^654^ EPHA2/LYN protein complex has responded to mechanical cues to regulate EMT and metastasis of cancer. The ligand-independent phosphorylation of ephrin receptor EPHA2 is triggered by high ECM stiffness through recruiting and activating the LYN kinase.^655^ EMT transcription factor TWIST1 is phosphorylated by LYN and releases TWIST1 to the nucleus, thereby facilitating EMT and invasion. More and more researchers started to regard the mechanical properties of microtubules and their dynamics as the major mechanism for tubulin.^656^ The change in microtubule properties affects the migration of cancer cells. A recent study showed that ECM stiffness-activated of glutamine catabolism can induce microtubule glutamylation to stabilize the MT network, thereby promoting cancer aggressiveness.^657^

For cancer dissemination, blood, and lymphatic circulations are important factors contributing to the efficiency of cancer cell transit from the primary tumor, metastasis, and extravasation.^658^ In the circulatory system, flow rates and FSS affect the survival of cancer cells. After being exposed to FSS in the circulatory system, anti-anoikis properties of the circulating tumor cells (CTCs) can be improved.^659^ Specific circulatory transition phenotype cancer cells can survive prolonged FSS and lead to cancer dissemination with a shorter survival.^660^ It has been demonstrated that low FSS can activate β1 integrin trafficking through cytoskeleton reconstruction and ROCK/histone deacetylase 6 (HDAC6)-mediated deacetylation of MT.^661^ In addition, intratumor pressure, ECM compression, and lack of functional lymphatic vessels can all lead to an increase in HP.^662^ The increased ECM macromolecules also contribute to elevated ECF elevation, which enhances cell migration and cancer dissemination through NHE1/RhoA/TRPV4-dependent signaling.^163^

#### Mechanisms of anti-cancer therapy resistance induced by mechanical cues

Cellular mechanotransduction in TME are critical in cancer immunotherapy and contribute to immunotherapy resistance.^663^ Mechanical cues lead to immunotherapy resistance by being engaged in the anti-tumor immunity cascade.

The ECM stiffness in the tumor is often 5–10 times that in normal tissue. For example, the normal stiffness of breast tissue is 0.4–2.0 kPa but increases to 12.0 kPa for breast cancer.^664^ Notably, the stiffness of a tumor changes at different tumor stages. For example, the elasticity of stage III lung adenocarcinoma is higher than that of the stage II.^665^ Currently, numerous studies have focused on the impact of ECM stiffness on cancer immunotherapy. PD-L1 overexpression is an adaptive immune resistance strategy for immune surveillance escape of cancer cells. It has been demonstrated that lung cancer cells cultured on a 25 kPa medium exhibited an increased expression of PD-L1 compared with those cultured on a softer medium, possibly due to F-actin polymerization.^666^ Furthermore, high ECM stiffness in TME will also affect immune cell behaviors in the immunotherapy against cancer. In response to ECM stiffness, the natural killer (NK) cells lyse cancer cells to release antigens. Dendritic cells (DCs) and macrophage cells are transformed into immunosuppressive phenotypes, which lead to immunotherapy resistance. ECM stiffness also triggers DCs differentiation and maturation.^667^ The migration of immune DCs (iDCs) through ECM depends on integrin-based adhesion structure. C-type lectin receptors (CLRs), which are found on the surface of iDCs, are downregulated by the stiff ECM in TME to prevent it from binding with internalized antigens. On the other hand, the degradation of ECM is essential for the migration and accumulation of NK cells.^668^ Elevated ECM stiffness breaks the balance between NK cell secretions of TIMP and MMP, thereby deterring NK cells to proceed with tumor-killer functions.^663^ In addition, tumor-associated macrophages (TAMs) are major immune cells in TME. Through a ROCK-independent, podosome-dependent mesenchymal migratory mechanism, the stiff ECM facilitates the transition of TAMs from an M1 to an M2 phenotype. The damage of phagocytosis and the migration ability of TAMs leads to immunotherapy resistance to cancer. For example, macrophage cells are activated by ECM stiffness via RhoA/ROCK signaling to promote the secretion of inhibitory immune factor IL-10. Then M2 phenotype macrophage cells are up-regulated, contributing to immunoresistance.^669,670^ However, the regulatory mechanisms between immune cells and ECM stiffness in immunotherapy resistance against cancers are still understudied.

Apart from ECM stiffness, other mechanical cues, such as blood or lymphatic vascular FSS in TME, can affect tumor cell biology. The rapidly structured and complicated vessels, as well as the compression of vessels, increase the geometric resistance to flow. Immunotherapy resistance is primarily brought on by high vascular FSS, which reduces T cell extravasation and immune monitoring for cancer cells.

Cancer stem cells (CSCs) are specific cancer cells that are characterized by self-renew and chemoresistance.^671^ Recently, EMT has been found to contribute to the generation of CSCs. During EMT, the phenotypes of cancer cells transform from epithelial properties (i.e., apical-basal polarity and cell junctions) to mesenchymal properties (i.e., invasion and migration),^672^ which reduce adhesion and connection of cells and facilitate cancer metastasis. More importantly, mechanical cues are critical factors contributing to the EMT of cancer cells. On one hand, mechanical cues directly drive the EMT process via mechanosensitive effectors (i.e., Piezo, YAP/TAZ, and integrin). For example, YAP promotes the expression of EMT-associated genes to facilitate cancer cell differentiation into CSCs.^673^ On the other hand, CAFs and TAMs in the CSCs can secret TGFβ to trigger EMT.^674^ Researchers have found that EMT is a developmental process being exploited by tumor cells. During this process, Axl kinase expression is critical in cancer metastasis.^675^ The inhibition of Axl can significantly reduce the EMT level, attenuate metastasis, and increase overall survival in breast cancer.^676^ Several clinical trials are ongoing in anti-cancer therapies targeting Axl-induced EMT, which can be driven by mechanical cues (Table 4).

**Table 4:** Clinical trials of anti-cancer therapies targeting Axl

Methods of drug application	Disease type	Phase	Current status	ClinicalTrials.gov identifier
Bemcentinib (before surgery); Bemcentinib (after surgery)	Brain and central nervous system tumors	1	Active	NCT03965494
Bemcentinib; docetaxel	Non-small cell lung cancer	1	Active	NCT02922777
Bemcentinib; pembrolizumab	Triple-negative breast cancer; inflammatory breast cancer stage IV	2	Terminated	NCT03184558
Bemcentinib; cytarabine; decitabine	Acute myeloid leukemia; myelodysplastic syndromes	1/2	Active	NCT02488408
Bemcentinib; pembrolizumab	Lung cancer metastatic; NSCLC stage IV; adenocarcinoma of lung	2	Completed	NCT03184571
Bemcentinib+pembrolizumab; Bemcentinib+dabrafenib and trametinib; pembrolizumab; dabrafenib and trametinib	Melanoma	1/2	Active	NCT02872259
Erlotinib; bemcentinib	Non-small cell lung cancer	1/2	Completed	NCT02424617
Bemcentinib	Acute myeloid leukemia; high-risk myelodysplastic syndrome; low-risk myelodysplastic syndrome	2	Completed	NCT03824080
Rucaparib; abemaciclib; pembrolizumab and bemcentinib	Mesothelioma, malignant	2	Active	NCT03654833

*Bemcentinib (BGB324)* a highly selective inhibitor of Axl

## Conclusions and perspectives

Based on the advanced research, the pivotal roles of cellular mechanotransduction in health and diseases are identified in this review. Furthermore, important effectors (i.e., Piezo, integrin, TRPV, and YAP/TAZ) and signaling pathways (i.e., TGFβ/Smad, JAK/STAT, Wnt/β-catenin, ERK1/2, and RhoA/ROCK) responding to mechanical stimulation have been summarized. The comprehensive review of cellular mechanotransduction in biological processes shall assist us in finding mechanisms and therapeutic targets associated with mechanical cues.

Following the development of scRNA-seq, researchers have identified different subtypes of fibroblast, which provides opportunities for precise inhibition of fibrogenic fibroblast.^677^ Researchers found that diverse subtypes of fibroblasts distribute differently, which function differently as well. Intriguingly, the tissue-specific fibroblast transformed to myofibroblast unexhaustedly. Hence, we speculate that mechanical cues in tissues may drive the migration of stem cells and storage fibroblasts. In addition, during the wound healing process, myofibroblasts aggregate at the frontier location of the wound with high tension and ECM with high elasticity deposition. Meanwhile, the fibroblasts with collagen enrich at the bottom of the wound.^678^ Hence, by learning the track of (myo)fibroblast in the tissue fibrosis, we may be able to detect a potential therapeutic target that does not affect the function of normal fibroblast.

Recently, the adeno-associated virus (AAV)-mediated gene therapy aroused the attention of researchers for its high safety, low immunogenicity, and long-term efficacy.^679–684^ With designed promoters, AAV targets specific tissues or organs and inhibits fibrosis. AAV serotype2 vector with miR19b transgene targeting collagen α I promoter was applied in the male Sprague Dawley rat hepatic fibrosis mode, and hepatic injuries were reduced in the treated group.^685^ Hence, AAV therapy may be a potential treatment targeting fibrosis in the future.

Besides, attention has been paid to the relationship between cellular mechanotransduction and stem cells in regenerative medicine.^686^ Mechanical cues can regulate stem cell differentiation for potential regenerative therapies for fibrotic diseases.^687^ Mechanical stress can also modulate stem cell differentiation to cardiovascular cell types,^688^ mechanical forces improve myocardium regeneration, and mechanical stretch can promote stem cell migration.^689^

A recent study suggested that mechanosensitive metabolism played a vital role in cancer cell migration and metastasis.^690^ Interestingly, cooperatively migrating cells alter leaders dynamically to reduce the thermodynamic costs of invasion.^691^ Thermodynamic evidence provides novel insights into the association between mechanotransduction and cancer cellular energy metabolism during migration, which indicates that cancer cell migrates through the minimal energy intake pathway. These findings provide novel ideas for preventing cancer dissemination. In addition, for the precise therapies of cancer, biomechanical cues are regarded as a novel strategy to reverse cancer immunotherapy resistance. Further research is warranted to investigate the mechanisms between mechanical cues and immunotherapy resistance. The investigation targeting mechanical properties of the tumor will provide approaches on the restriction of cancer cell behaviors.

Mechanobiology is also being widely investigated in 2D configurations. However, limited studies of cell-ECM matrix forces have been performed in 3D systems. Although the 2D system is considered the foundation of mechanical cues-associated pathophysiology, the 3D platform is more similar to the microenvironment of the human body.^692,693^ A recent study has reported a novel 3D platform consisting of a well-defined synthetic hydrogel system and 3D traction force microscopy to evaluate the matrix environment and force response. The platform provides a novel insight into cell behaviors in mechanotransduction in a 3D system.^694^ Hence, mechanical transduction in the 3D microenvironment needs more attention in the future.

In summary, as an essential factor contributing to health and diseases in organisms, cellular mechanotransduction widely affects tissue homeostasis, fibrotic diseases, tumorigenesis, metabolism, and others. Therefore, a broader overview of mechanical cues is a great challenge for researchers. This current review presents the mechanisms of mechanical cues-associated pathophysiological processes in organisms and sheds light on the therapeutic targets of multiple diseases.
